# Telomemore enables single-cell analysis of cell cycle and chromatin condensation

**DOI:** 10.1093/nar/gkaf031

**Published:** 2025-01-29

**Authors:** Iryna Yakovenko, Ionut Sebastian Mihai, Martin Selinger, William Rosenbaum, Andy Dernstedt, Remigius Gröning, Johan Trygg, Laura Carroll, Mattias Forsell, Johan Henriksson

**Affiliations:** Laboratory for Molecular Infection Medicine Sweden (MIMS), Umeå University, Biomedicinbyggnaden 6K och 6L, Umeå universitetssjukhus, 901 87, Umeå, Sweden; Umeå Centre for Microbial Research (UCMR), Universitetstorget 4, 901 87, Umeå, Sweden; Department of Molecular Biology, Umeå University, Biomedicinbyggnaden 6K och 6L, Umeå universitetssjukhus, 901 87, Umeå, Sweden; Laboratory for Molecular Infection Medicine Sweden (MIMS), Umeå University, Biomedicinbyggnaden 6K och 6L, Umeå universitetssjukhus, 901 87, Umeå, Sweden; Umeå Centre for Microbial Research (UCMR), Universitetstorget 4, 901 87, Umeå, Sweden; Department of Molecular Biology, Umeå University, Biomedicinbyggnaden 6K och 6L, Umeå universitetssjukhus, 901 87, Umeå, Sweden; Industrial Doctoral School, Umeå University, Umeå, Sweden; Laboratory for Molecular Infection Medicine Sweden (MIMS), Umeå University, Biomedicinbyggnaden 6K och 6L, Umeå universitetssjukhus, 901 87, Umeå, Sweden; Umeå Centre for Microbial Research (UCMR), Universitetstorget 4, 901 87, Umeå, Sweden; Department of Molecular Biology, Umeå University, Biomedicinbyggnaden 6K och 6L, Umeå universitetssjukhus, 901 87, Umeå, Sweden; Department of Chemistry, Faculty of Science, University of South Bohemia, Ceske Budejovice 37005, Czech Republic; Department of Molecular Biology, Umeå University, Biomedicinbyggnaden 6K och 6L, Umeå universitetssjukhus, 901 87, Umeå, Sweden; Department of Clinical Microbiology, Umeå University, Biomedicinbyggnaden 6M, Umeå universitetssjukhus, 901 87, Umeå, Sweden; Department of Clinical Microbiology, Umeå University, Biomedicinbyggnaden 6M, Umeå universitetssjukhus, 901 87, Umeå, Sweden; Department of Chemistry, Umeå University, Linnaeus väg 10, Umeå universitet, 901 87, Umeå, Sweden; Sartorius Corporate Research, Östra Strandgatan 24, 903 33, Umeå, Sweden; Laboratory for Molecular Infection Medicine Sweden (MIMS), Umeå University, Biomedicinbyggnaden 6K och 6L, Umeå universitetssjukhus, 901 87, Umeå, Sweden; Umeå Centre for Microbial Research (UCMR), Universitetstorget 4, 901 87, Umeå, Sweden; Department of Clinical Microbiology, Umeå University, Biomedicinbyggnaden 6M, Umeå universitetssjukhus, 901 87, Umeå, Sweden; Integrated Science Lab (IceLab), Umeå University, Naturvetarhuset, Universitetsvägen, 901 87, Umeå, Sweden; Department of Clinical Microbiology, Umeå University, Biomedicinbyggnaden 6M, Umeå universitetssjukhus, 901 87, Umeå, Sweden; Laboratory for Molecular Infection Medicine Sweden (MIMS), Umeå University, Biomedicinbyggnaden 6K och 6L, Umeå universitetssjukhus, 901 87, Umeå, Sweden; Umeå Centre for Microbial Research (UCMR), Universitetstorget 4, 901 87, Umeå, Sweden; Department of Molecular Biology, Umeå University, Biomedicinbyggnaden 6K och 6L, Umeå universitetssjukhus, 901 87, Umeå, Sweden; Integrated Science Lab (IceLab), Umeå University, Naturvetarhuset, Universitetsvägen, 901 87, Umeå, Sweden

## Abstract

Single-cell RNA-seq methods can be used to delineate cell types and states at unprecedented resolution but do little to explain why certain genes are expressed. Single-cell ATAC-seq and multiome (ATAC + RNA) have emerged to give a complementary view of the cell state. It is however unclear what additional information can be extracted from ATAC-seq data besides transcription factor binding sites. Here, we show that ATAC-seq telomere-like reads counter-inituively cannot be used to infer telomere length, as they mostly originate from the subtelomere, but can be used as a biomarker for chromatin condensation. Using long-read sequencing, we further show that modern hyperactive Tn5 does not duplicate 9 bp of its target sequence, contrary to common belief. We provide a new tool, Telomemore, which can quantify nonaligning subtelomeric reads. By analyzing several public datasets and generating new multiome fibroblast and B-cell atlases, we show how this new readout can aid single-cell data interpretation. We show how drivers of condensation processes can be inferred, and how it complements common RNA-seq-based cell cycle inference, which fails for monocytes. Telomemore-based analysis of the condensation state is thus a valuable complement to the single-cell analysis toolbox.

## Graphical Abstract

**Graphical Abstract:**
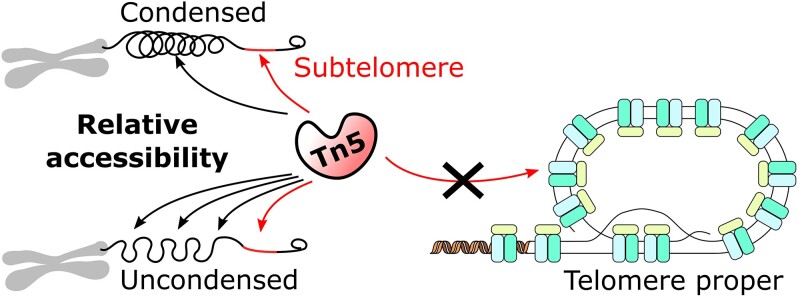

## Introduction

The maintenance of genome integrity is an essential biological process, as errors can lead to loss of genes or cancer. The end of chromosomes (telomeres) need special treatment, as the typical DNA replication process results in the loss of 50–100 bp per cell division [1]. To overcome this, various mechanisms exist to extend the telomere, which in e.g. humans and mice, mainly consists of 6 bp tandem repeats of TTAGGG, protected by the Shelterin complex [2]. Because short telomeres can indicate or be the cause of over 13 pathogenic conditions (from liver fibrosis to cancer [3]), there is considerable interest in measuring the length of this region. Several methods, such as qPCR (quantitative PCR), terminal restriction fragments Southern blot (TRF), fluorescence *in situ* hybridization (FISH), and long-read sequencing have been developed [4, 5]. There is however a need to study telomere length also in individual cells and correlate it with other features of interest. In cancer cells, for example, telomeres can be both shorter and longer than in normal cells [6]; such single-cell differences may not be detected if measured using “bulk” methods, which provide an average over many cells. Furthermore, telomere length varies across cell types, and without an understanding of what cell is being studied, the length may have little meaning. In line with this reasoning, telomere length from bulk cancer measurement is not considered a good biomarker [4].

Single-cell RNA-seq is the current state-of-the-art tool to understand heterogeneity of cells in a tissue. Limited efforts have been made to also include telomere length measurement, but the largest study to date only measured telomere lengths in 242 cells, likely because it was performed in low-throughput and costly multi-well plates [7]. As contemporary RNA-seq-only single-cell atlases can easily measure 100 k to 1 M cells, this begs for a new single-cell approach to simultaneously measure cell state and telomere length.

**Figure 1.**
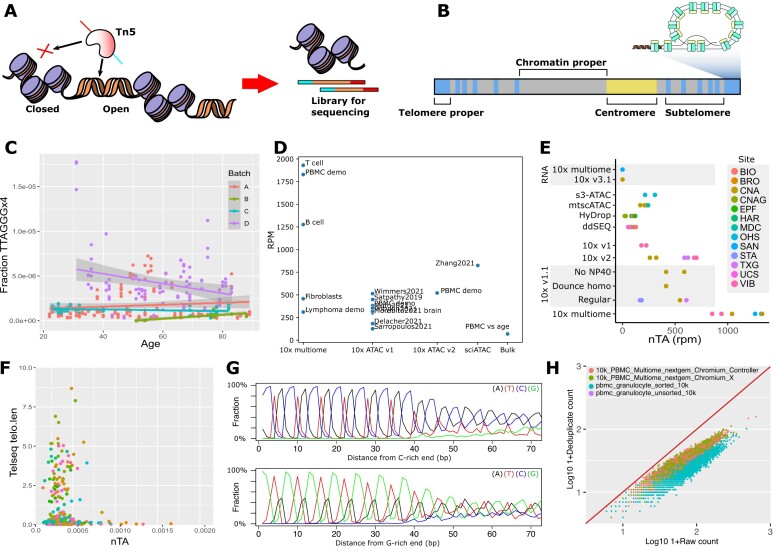
Telomere repeat k-mer-based counting of ATAC-seq libraries does not correlate with telomere length measurement. (**A**) ATAC-seq is a modern method in which accessible chromatin is “tagmented,” i.e. fragmented by the enzyme Tn5, which also adds adapters for sequencing. These fragments are normally used to analyze enhancers. (**B**) Components of the genome, as discussed in the paper. Tandem repeats of TTAGGG are also present in the subtelomere, which does not have a clear end. We refer to the outermost tandem repeat as the “telomere proper,” and what is neither telomere, subtelomere, nor centromere, as “chromatin proper.” (**C**) Normalized abundance of TTAGGG (nTA) across PBMC ATAC-seq datasets does not correlate with age. The common method to infer telomere length from WGS data does thus not seem to work for ATAC-seq. The batches A–D are described in methods. (**D**) There is a large variation in nTA across different datasets and ATAC-seq methodologies. The B cell and fibroblast multiome datasets are included in this study; the T-cell dataset is in a separate publication [101]; RPM, repeats per million. (**E**) Comparison of nTA in PBMCs across multiple technologies [102]. (**F**) The ATAC-seq nTA does not show correlation to telomere length in TCGA cancer samples. The cancer type does not appear to influence the correlation (three outliers cropped; legend in Supplementary Fig. S2B). (**G**) Average motif of telomere-like reads, after alignment by GC content. (**H**) Deduplication shows that motif-containing reads have large sequence diversity.

ATAC-seq (Assay for Transposase-Accessible Chromatin using sequencing) is a widely used method for characterizing the epigenetic state by finding open chromatin regions [8]. ATAC-seq is based on the enzyme Tn5 fragmenting and tagging (“tagmenting”) open chromatin, which is then sequenced (Fig. 1A). The accessibility of regions can then be compared, and in particular, transcription factor (TF) activity can be quantified based on motif presence in accessible regions [9]. Thus, potential upstream regulators of different cell types and fates can then be pinpointed. Gene expression levels can also be approximated from the accessibility of their transcriptomic start site (TSS). With the goal of generating novel readouts from ATAC-seq data, we discovered telomere-like reads. We hypothesized that potential tagmentation of telomeres could also be used for telomere length measurement, as longer telomeres could possibly result in more ATAC-seq reads. Thus, we analyzed both public and novel data to validate this hypothesis.

Here, we show that single-cell ATAC-seq is unsuitable for inferring telomere length, largely because the telomere proper appears protected from Tn5 transposition. Instead, we show that, in most cases, presence of telomere-like reads rather indicate general condensation of the chromatin, as they are of mostly subtelomeric origin, and the subtelomere becomes relatively more accessible during condensation (Fig. 3I). While the cell cycle stage can be inferred from RNA-seq, we claim that ATAC-seq sometimes is a more direct assessment of the entry to, and exit from, mitosis. Further analysis of the centromere may also help separate between early and late G_1_-phase. We provide a tool (Telomemore, latest version maintained at https://github.com/henriksson-lab/telomemore.java) to assess chromatin condensation. This is validated using a new long-read atlas of Tn5 insertions, as well as by a convolutional neural network (CNN) trained to approximately infer the origin of short reads. We provide examples, which showcase how metrics reported by Telomemore can aid in the interpretation of single-cell datasets, including (i) an analysis of TFs controlling chromatin condensation, as well as (ii) how CDKN1C seems to lock atypical monocytes in G_1_-phase. Finally, we provide a new single-cell atlas of human tonsil B cells and show that somatic hypermutation (SHM) can be contextualized in terms of chromatin condensation. By providing precomputed chromatin condensation scores for several public single-cell datasets, we envision that further insights into the regulation of overall chromatin state will be unlocked.

## Materials and methods

### Acquisition of public single-cell data

Suitable datasets for reanalysis were mainly found from the 10× Genomics publication database (https://www.10xgenomics.com/datasets). To determine if sequencing reads were available, datasets were manually inspected using the National Center for Biotechnology Information (NCBI) Sequence Read Archive (SRA) online preview function (https://www.ncbi.nlm.nih.gov/sra). Whenever datasets had missing cell barcodes (“technical reads”), we instead attempted to obtain the complete original data using the SRA cloud retrieval function. We note that in SRA, cell barcode reads are inconsistently denoted as “biological” or “technical.” To ensure that we obtained the cell barcodes, we retrieved the data using the command “fasterq-dump SRRxxx -e 10 -v –include-technical –split-files.”

Furthermore, we processed and broadly analyzed the following datasets: Delacher2021 [10] (GSE156112), Jain2021_pbmc [11] (E-MTAB-11225, E-MTAB-11226), Jain2021_thymus [11] (E-MTAB-9828, E-MTAB-9840), Lyu2021 [12] (GSE183684), Morabito2021 [13] (GSE174367), Sarropoulos2021 [14] (E-MTAB-9765), Satpathy2019 [15] (GSE129785), Taavitsainen2021 [16] (GSE168667), Wimmers2021 [17] (GSE165904), Ziffra2021 [18] (GSE163018), Kinoshita2021 [19] (GSE131549), Qiangli2021 [20] (GSE178551), and Zhang2021 [21] (GSE184462).

The Satpathy2019 GEO dataset GSE129785 was not compatible with ArchR. Also, as cell barcodes were missing in the SRA upload, SRA cloud delivery was used. BAM files were converted to FASTQ using bamtofastq (https://github.com/10XGenomics/bamtofastq/), and then realigned using CellRanger. Similarly, we solved issues with the download of Ziffra2021 and Delacher2021 using the cloud delivery service.

For multiome mouse embryo data (Argelaguet2022) [22], rather than realign the raw files (GSE205117), we used the processed count and ATAC fragment files provided via FTP (https://github.com/rargelaguet/mouse_organogenesis_10x_multiome_publication).

### Generation of a tonsillar B-cell multiome atlas

The research was carried out according to The Code of Ethics of the World Medical Association (Declaration of Helsinki). Ethical permits were obtained from the Swedish Ethical review authority (number: 2016/53–31), and all samples were collected after receiving informed consent from patients or patients’ guardians. Briefly, tonsillar cell suspensions were prepared by tissue homogenization in RPMI-1640 medium and passed through a 70 μm cell strainer. Red blood cells were lysed using BD PharmLyse lysis buffer according to the manufacturer’s instructions. All cell suspensions were frozen in fetal bovine serum (FBS) (Gibco) with 10% DMSO (Dimethyl sulfoxide) and stored in liquid N_2_.

B cells from tonsils of five individuals were enriched using negative selection magnetic beads (EasySep Human B Cell Isolation kit, Stemcell technologies, #17954). They were then pooled to avoid batch effects, with an average viability per donor of 92% (SD: 1.5%).

Enriched B cells were washed in ice-cold ATAC-seq resuspension buffer (RSB), (10 mM Tris, pH 7.4, 10 mM NaCl, 3 mM MgCl_2_), spun down, and resuspended in 100 ml of ATAC-seq lysis buffer (RSB plus 0.1% NP-40 and 0.1% Tween-20, Thermo Fisher). Lysis was allowed to proceed on ice for 5 min, then 900 ml of RSB was added before spinning down again and resuspending in 50 ml of 1× Nuclei Resuspension Buffer (10× Genomics). To assess nuclei purity and integrity after lysis, nuclei were stained with Trypan Blue (15250061, Thermo Fisher Scientific) and DAPI (4′,6-diamidino-2-phenylindole, D1306, Thermo Fisher Scientific), according to the manufacturer’s recommendation. If necessary, cell concentrations were adjusted to equal ratios (per donor) prior to starting single-cell GEM (Gel Beads in emulsion) droplet generation with the ATAC-seq NextGEM Kit (10× Genomics). Briefly, nuclei were incubated in a transposition mix. Transposed nuclei were then loaded into a Chromium Next GEM Chip J. A total of 9000 nuclei were loaded per lane, with a target recovery of 5500 (doublet rate 4%–4.8%). After generation of GEM emulsions, we performed reverse transcription, cDNA (complementary DNA) amplification and library indexing according to manufacturer specifications (*Chromium Next GEM Single Cell Multiome ATAC + Gene Expression*, CG000338 Rev A, 10× Genomics, September 2020).

### Generation of a colonic fibroblast multiome atlas

Human colonic fibroblasts (CRL-1459, ATCC) were maintained in Eagle’s Minimum Essential Medium (EMEM; 30–2003, ATCC) supplemented with 10% of FBS (10270–106, Gibco, Thermo Fisher Scientific) and 10 μg/ml ciprofloxacin (17850, Sigma‐Aldrich Merck), as described before [23]. A single-cell multiome library was generated analogously to B-cell libraries, with lysis continuing for 5 min, and 3000 nuclei loaded into the Chromium instrument. Passages 10 and 15 were collected, but only passage 10 was included here due to (i) large differences in telomere-like read abundances, and (ii) the latter passage seemed to enter terminal differentiation. The heterogeneity was too complex for the purposes of our validation. Raw data have been submitted to ArrayExpress (E-MTAB-14220).

### Telomere *k-*mer motif data analysis in short-read data

The Telomemore pipeline was first implemented in Python, and later in Java for performance and for the ease of supporting multiple input file formats in an object oriented manner. To detect telomeric reads, CCCTAA and TTAGGG-motif scanning was performed, either using (i) custom python scripts with FASTQ files as input or (ii) using the Telomemore software, which also supports BAM files as input and cell barcode assignments. At least three consecutive telomere motifs were required for a read to be counted, which was qualitatively motivated based on a histogram analysis, but has also been motivated previously based on comparison to the gold standard TRF [24].

To be able to compare the number of telomeric reads between libraries, we defined normalized telomere abundance (nTA) as the number of telomere-like fragments divided by the total number of reads:


\begin{equation*}{\rm nTA} = \frac{{{\rm Telomere }- {\rm like}\ {\rm reads}}}{{{\rm Total}\ {\rm number}\ {\rm of}\ {\rm reads}}}\end{equation*}

This measure takes sequencing depth into account (for a whole bulk ATAC library, or per single cell). For visualization, we used rank(nTA) to maximize contrast.

### Analysis of bulk aging PBMC ATAC-seq

We aligned the PBMC versus aging dataset [25] to the cellranger-arc-GRCh38-2020-A-2.0.0 reference genome using STAR v2.7.1.a. To find sources explaining the variation, we calculated the nucleosome signal from the histogram of mapped read lengths, computed by ATACFragQC v0.4.5. Furthermore, we qualitatively investigated all metadata available for each donor (e.g. by considering read length, as one batch in particular had a different read length; by considering telomere content, as telomere content was dramatically different for donors with high donor IDs; and by assessing variant telomere repeats [T**GGG]). Overall, we identified four batches A–D; Batch D contains samples with subject ID >260, C remainder with average read length >130, B remainder with read length >80, and batch A contains the rest of the samples. We then fitted telomere abundance versus age for each batch. We also attempted several variants of multilinear models, including covariates such as sex, nucleosome signal, batch, and number of centromeric reads (inferred by CNN model, described later). We further attempted to include the number of variant telomere repeats (TAAGGG, TTTGGG, etc.), inspired by previous work [26]. None of the aforementioned approaches resulted in a statistically significant or consistent correlation with age across batches.

The bulk peripheral blood mononuclear cell (PBMC) dataset was also used to study the binding preference motif of Tn5. Since short telomeric reads cannot reliably be aligned to the reference genome, a reference-free approach was used to reorient the reads (Supplementary Fig. S1D). In short, if the *G*% content was below 50% on the 5′ side, the forward (R1) and reverse (R2) reads were selectively reverse complemented and swapped. A custom R script was then used to estimate base frequencies at each position.

### Inference of the approximate origin of reads using a convolutional neural network

A convolutional neural network was implemented in PyTorch with the aim of predicting the average position of a 50 bp sequence from the telomere. The distance is defined as 0 for any sequence within the telomere proper. The centromere region was taken from the T2T (Telomere-to-telomere) reference genome using the associated repeat masker GTF-file, while the chromatin proper is defined as the remainder, up to 10 Mbp from the end of each chromosome. The sequence was then extracted using getfasta from Bedtools 2.30.0 [27].

To ensure that the natural human variability of the subtelomere is handled by the algorithm, nanopore data of the telomeres of 18 random individuals were analyzed [28, 29] (IDs in dataset: 3048691–3048709). The sequence of the biotin adapter (CCCTCCGATA) ligated to the telomeres was trimmed from the beginning of each read. BAM files were read by invoking “samtools view” in a pipe using the Python subprocess library. Reads <8 kb were omitted. To define the end of the telomere proper, the read sequence was repeatedly scanned for TAACCC within 0 to 18 bp from the current position. When no more matches are found, the current position is set at the start of the subtelomere. The results were deemed similar to manual curation.

The philosophy of the network was to have few parameters to try and avoid overfitting, but still acceptable performance for the purpose. Each base of ATCGN was one-hot encoded for the input. Several designs were qualitatively evaluated. The final design was as follows: a convolutional layer #1 (kernel size is 6 and 20 filters), ReLU (rectified linear unit), convolutional layer #2 (kernel size is 6 and 20 filters), ReLU, and a dense layer. The kernel size was selected to aid the network in detecting the telomere hexamer sequence. A total of 500 000 random centromere/chromatin sequences were used for training, along with 500 000 random sequences from the telomere nanopore data. The reverse complements of the sequences, computed using Biopython [30], were also included. The data were split 80/20 into training and test datasets. The training error was defined as the mean squared error of the predicted log(distance) from the telomere. The model was trained for 200 epochs although 100 epochs seemed sufficient. No overfitting was noticed based on test data predictions.

The precise log(distance) prediction was not used for comparison with nTA, as further hyperparameter optimization and deep validation are likely required. Instead, predictions were binned to be within the distances of each respective training dataset, and reads with distance = 0 were considered subtelomeric (as such reads are very sparse and long-read data suggest them to seldom be from the telomere proper).

### Analysis of TCGA cancer data

All 410 ATAC-seq libraries were downloaded from the TCGA (The Cancer Genome Atlas), effectively from a single study [31]. Using the TCGA portal, the corresponding whole genome sequencing (WGS) samples were located. Telomere lengths were then obtained from a previous analysis [32] that used TelSeq [24]. Because ATAC nTA is affected by cell type, each cancer type was analyzed separately. Qualitatively, no correlation was found.

### Generation of long-read WGS libraries after Tn5 transposition

CCRF-CEM (CCL-119, ATCC) and Jurkat cells, both of T-cell origin, were cultured in RPMI (Roswell Park Memorial Institute) 1640 medium (cat no. 21870076, Thermo Fisher Scientific) with 10% bovine serum (cat #A3294-10G, Sigma-Aldrich) 1% 100× penicillin–streptomycin–glutamine.

For transpososome assembly, the preanealed cargo 250 nM duplex PAGE (polyacrylamide gel electrophoresis)-purified oligos were ordered from IDT (Coralville): sense (5′-CTGTCTCTTATACACATCTACGCGGTGGACAAAAAATTTCATTTGGAACTAGATTTGACCTCAGCTTCAATGCCAGAGATGTGTATAAGAGACAG) and antisense (5′-CTGTCTCTTATACACATCTCTGGCATTGAAGCTGAGGTCAAATCTAGTTCCAAATGAAATTTTTTGTCCACCGCGTAGATGTGTATAAGAGACAG). Cargo oligos of 95 bp were created with Mosaic ends (ME) [33] and with a restriction site for BbvCI. Such length was previously found in one of the potential optimal windows for binding Tn5 transposase [34]. The body of the cargo was modified from the parts of the Tn5 transposon (GenBank KF813062.1) that were not creating the hairpin according to the folding tool Mfold [35]. The cargo concentration was adjusted to 67 μM in the buffer (2 mM DTT (dithiothreitol), 771 mM NaCl, 44.7 mM Tris–HCl, pH 7.5, 10% glycerol) and stored at −20°C. Tn5 _E54K, L372P_ manufactured and purified at EMBL Heidelberg [36] was removed from −80°C and thawed. Glycerol concentration was adjusted to 50% and stored for a few weeks at −20°C. Tn5 was assembled with cargo oligonucleotides at equal molar. The mixture was incubated at room temperature, shaking at 400 revolutions per minute (rpm) for 1 h. ATAC-seq [37] was modified to enable long-read analysis. Briefly, CCRF-CEM (ECACC 85112105) and Jurkat cells were grown overnight, and 100 000 cells were were harvested at 500 × *g* at 4°C for 5 min. Cell pellets were resuspended in 50 μl of lysis buffer (10 mM Tris–HCl, pH 7.4, 10 mM NaCl, 3 mM MgCl_2_, 0.1% NP-40, 0.1% Tween, 0.01% Digitonin) and kept for 4 min on ice, after which 500 μl of wash buffer (10 mM Tris–HCl pH 7.4, 10 mM NaCl, 3 mM MgCl_2_, 0.1% Tween) was added and gently resuspended by pipetting and centrifuged at 500 × *g* at 4°C for 15 min. The nuclei pellets were resuspended in the transposition reaction mix (TD buffer with 0.1% Tween, 0.01% Digitonin, containing Tn5 transposase). The final concentrations of Tn5 in the reaction were 50 ng/μl. Reactions were incubated for 2 h at 37°C shaking at 700 rpm. The transposed gDNA (genomic DNA) was purified using the Monarch Genomic DNA Purification Kit (catalog no. T3010S, New England Boilabs), and length and quality were accessed by gel. The estimated amount of inserts was assessed by qPCR with primers against the insert (Forward: 5′-ACGCGGTGGACAAAAA; Reverse: 5′-CTCTTATACACATCTCTGGCATT); inserts were also checked by polymerase chain reaction (PCR) with primers that go outwards the insert (Forward: 5′-TCAATGCCAGAGATGTGTATAA; Reverse: 5′-CACCGCGTAGATGTGT). Samples from the few repetitions were pooled and sent for PacBio sequencing (SciLifeLab, NGI Sweden).

### Analysis of long-read WGS libraries after Tn5 transposition

Alignment was performed using pbmm2 v.1.13.0 (https://github.com/PacificBiosciences/pbmm2), a wrapper for minimap2 [38], against the T2T reference genome, which was indexed using default parameters. Pileups were produced using bamCoverage 3.5.1 from deepTools [39] and visualized using IGV 2.16.0 [40]. A custom R script was used to detect the Tn5 insert sequence and perform statistical analyses. Tn5-insert containing reads were gathered and motifs were visualized using ggseqlogo [41].

### Statistical analysis of monocytes

We compared rank(nTA) for the four PBMC batches we had access to (Supplementary Fig. S4B and C). If all cells are treated equally, then nTA is different between classical and nonclassical monocytes with *P* < 2.2e16 (*t*-test). Since multiple batches are present, we also statistically compared the averages. All batches result in *P*< .064. Library #4 (pbmc_granulocyte_unsorted_10k) appears to not capture differences in nTA; removing this library would result in *P*< .038.

### FACS cell cycle analysis of monocytes

The research was carried out according to the Code of Ethics of the World Medical Association (Declaration of Helsinki), and an ethical permit was obtained from the Swedish Ethical Review authority (#2016/53–31). Blood samples were obtained from human healthy male adults, ages 18–30. PBMCs were isolated by gradient density centrifugation using Ficoll-Paque PLUS (Cytiva, 17144002) and stored at −150°C. Thawed cells were counted and brought to 10^6^ cells per tube in 100 μl of fluorescence-activated cell sorting (FACS) staining buffer (0.1% sodium azide and 2% FBS in PBS). The cells were then stained for 30 min at 4°C in the dark with antihuman antibodies specific for anti-CD14 [APC/Cyanine-7] (301819, BioLegend) and anti-CD16 [fluorescein isothiocyanate (FITC)] (360715, BioLegend). For cell cycle analysis, cells were additionally stained by Hoechst 33258 (94403, Sigma-Aldrich) at 10 μg/ml for 30 min at room temperature and additionally stained for viability discrimination by 5 PI μg/ml (P3566, Thermo Fisher Scientific). Samples were washed, resuspended in FACS staining buffer, and taken for FACS analysis. At least 30 000 gated monocytes were acquired in a 9-color BD FACSMelody flow cytometer (BD Biosciences) using FACSDiva software (BD Biosciences) and analyzed using FlowJo software (TreeStar, v10.9, Ashland, OR). Gating of the monocytes was done excluding the doublets and without the exclusion of dendritic cells. The cell cycle of the monocytes was assessed by fitting a mixture model using the FlowJo Cell Cycle function [42]. Gated classical and nonclassical monocytes were sorted in the FACS tubes, pelleted by centrifugation, and imaged at 40× magnification with a Zeiss AxioPlan 2 in the bright field for the morphology.

### General analysis of single-cell data

Unless otherwise stated, all 10× Genomics single-cell ATAC-seq data were aligned using cellranger-atac-2.0.0. Multiome RNA + ATAC-seq datasets were aligned with cellranger-arc 2.0.0. Cell types were annotated according to markers from each respective source article, or annotation files as described for each dataset below. Our custom pipeline Telomemore was then used to count reads for each cell having at least three consecutive telomere motifs, as for the previous bulk analysis. Single-cell RNA-seq analysis was done using Seurat [11] and single-cell ATAC-seq analysis using Signac [43]. MACS2 was used for peak calling [44]. Dimensional reductions were done using UMAP (Uniform manifold approximation and projection) [45]. We refer to the provided R source code for the precise details of this analysis.

### Specific analysis of 10× genomics multiome PBMC/monocyte data

We obtained the 10× PBMC multiome PBMC demo dataset from www.10xgenomics.com [“Fresh Frozen Lymph Node with B-cell Lymphoma (14k sorted nuclei),” “10k Human PBMCs,” “Multiome v1.0, Chromium Controller,” and “10k Human PBMCs, Multiome v1.0, Chromium X”). Cell types were first predicted using SingleR [46], based on the DICE [47], HPCA [48], and Monaco [49] reference datasets. The final cell type annotation was performed using marker genes, applied to clustering by the Leiden algorithm. The nTA–gene correlations were computed on the clusters referenced in the text.

### Specific analysis of scGET-seq single-cell data

Along with FASTQ files for nTA computation, preprocessed tn5 and tnH count matrices were obtained from ArrayExpress (E-MTAB-10218, E-MTAB-10219, E-MTAB-9651, and E-MTAB-9659) and used for clustering. Routine scATACseq analysis was performed using Seurat/Signac. The ratio of TnH/Tn5 was then calculated and compared with nTA.

### Specific analysis of the Zhang2021 chromatin accessibility atlas

Besides GSE184462, the Zhang2021 atlas further depends on reuse of a pancreas dataset, GSE160472 [50], which we included. Privacy protected samples (human heart samples on dbGaP: phs001961, human islet samples on dbGaP: phs002204) were not included. Because the sciATACseq cell barcodes reside in the FASTQ name of the sequencing read and NCBI SRA strips the read names in their upload, we had to use GEO cloud delivery. Due to the size of the data, we did not perform alignment and *de novo* cluster assignment. Instead, existing cell type annotations were downloaded from https://data.mendeley.com/datasets/yv4fzv6cnm/1 (1B_Cell_metadata.tsv.gz).

To find motifs linked to telomere accessibility, a linear model was set up using Limma [51] with models as shown in the main figure. Motif scores were calculated using chromVar [9] through ArchR. CISBP was used as the motif database (http://cisbp.ccbr.utoronto.ca/) [52].

### Specific analysis of tonsillar B-cell multiome data

Library reads were aligned and aggregated using CellRanger ARC 2.0.0. The classification of TCRs (T-cell receptors) was done using TRUST4 [53]. The SNPs (single-nucleotide polymorphism) of the cells were extracted using cellSNP [54] and assigned to donors using Vireo [55]. No batch effects between donors were observed. The Vireo doublet score was used to filter out droplets. Cell types were predicted using SingleR [46], based on the HPCA (Human Primary Cell Atlas) [48] and Monaco [49] reference datasets. Furthermore, cell labels were transferred using Seurat from a previous single-cell RNA-seq-only tonsillar B-cell dataset [56], for qualitative comparison. Major axes [germinal center (GC)/non-GC and naive versus switched] aligned but precise clusters did not align satisfactorily. The dataset clusters, however, had similar topology if annotated based on the same marker genes. We did not find discrete clusters corresponding to “activated” nor “FCRL3^hi^.” Cells not of interest (T cells and dendritic cells) were present but ignored in this study. The marker gene CXCR4^hi^ was used as a marker for the dark zone (DZ). All highly varying genes and motif activities were correlated to nTA using Spearman’s method.

Because it has been reported that GC B cells, compared to naive and memory, are higher in TERT (telomerase reverse transcriptase) and have on average longer telomeres [57], we also investigated this alternative explanation for AICDA^hi^ cells being nTA^hi^. TERT is however upregulated in rather separate S-phase cells without effect on nTA. Telomeres have also been found to be 1.4 kb longer in naive T cells over memory T cells [58]; however, assuming an analogy for B cells, we cannot see such a trend on nTA in our data.

### Analysis of TERRA

For the purpose of analyzing TERRA (Telomeric Repeat-Containing RNA) in Parse Biosciences data, the raw data for their Evercode WT v2 PBMC dataset (https://www.parsebiosciences.com/datasets/performance-of-evercode-wt-v2-in-human-immune-cells-pbmcs/) was requested from customer support. The processed count table was downloaded from the website. Clustering was performed as usual, and the data subsetted to correspond to the monocytes in the 10× Genomics PBMC multiome dataset.

## Results

### The telomere proper is protected from transposition, making ATAC-seq unsuitable for telomere length inference

The telomere length has previously been inferred from WGS data [24,26,59]. All current computational methods are based on the classification of reads based on their content of telomeric motifs. The first implementation showed that three occurrences of TTAGGG in a read is sufficient to find a correlation with the mean length of terminal restriction fragments (mTRF), which is often considered the gold standard for telomere abundance measurement [24]. From a WGS library, it is only possible to compute the fraction of telomere-like reads (normalized telomeric abundance). To get actual telomere length, this must be multiplied by a constant fitted from matching mTRF data. A naive estimator of telomere length is thus:


\begin{eqnarray*} {\rm Telomere}\ {\rm length }&=& {\rm constant}*\ {\rm nTA},\ {\mathrm{with\ }}{\rm nTA} \nonumber\\ &=& \frac{{{\rm Telomere }- {\rm like}\ {\rm reads}}}{{{\rm Total}\ {\rm number}\ {\rm of}\ {\rm reads}}} \end{eqnarray*}

This equation assumes the simplified telomere model in (Fig. 1B), and we define the “telomere proper” to be near-pure repeats of TTAGGG. More recent telomere length estimation methods also account for telomere variant repeats (TVRs), sequences mainly present in the subtelomeric regions according to previous models [26]. While newer data complicates the binarized distinction between telomere and subtelomere, we will still assume this model to simplify the analysis and discussion.

Our initial idea was thus to estimate telomere length as for WGS, but using scATAC-seq data rather than scWGS, as scATAC-seq is easy to perform and such data is much more abundant. Because ATAC-seq does not randomly cover the genome and the absence of knowledge on its ability to target the telomeres, it was however not clear that this is possible.

To evaluate whether ATAC-seq data could be used to infer telomere length or not, we computed nTA for one of the largest bulk ATAC-seq datasets available, from PBMCs of 54 men and 66 women [25] (divided into three batches, analyzed separately to account for batch effects). However, unlike previous analyses using WGS data, we did not observe the expected decrease in ATAC telomere motif abundance with age (Fig. 1C). Looking for potential technical reasons for the lack of correlation, we found large differences in telomere-like fragment abundances across different ATAC-seq implementations (Fig. 1D and E). The abundance has increased in newer kits from 10 genomics, peaking in their multiome kit. Sample replicates have similar nTA, but there is considerable batch variation. However, we were unable to find a correlation between nTA and age, even when accounting for common ATAC-seq quality control metrics (such as nucleosome signal and percentage of mitochondrial reads; Supplementary Fig. S1A–C). To further confirm this lack of correlation, we also reanalyzed ATAC-seq of cancer samples from TCGA, comparing them to the telomere length inferred via WGS. We found no correlation between nTA and telomere length, even when accounting for differences in tissue origin (Fig. 1F and Supplementary Fig. S2).

One possible explanation for the lack of correlation is that the telomere-like reads may not be of telomeric origin. While most of the detected reads had similar sequences and appeared to be tagmented at the same position (Fig. 1G), there was still enough sequence variation to perform deduplication (Fig. 1H). This does not appear compatible with a telomeric origin, as the telomere is almost perfectly repetitive and thus the reads should be highly similar. However, the large amount of telomere-like repeats in the genome, combined with the use of short read sequencing, prevents detection of the origin by means of alignment.

**Figure 2.**
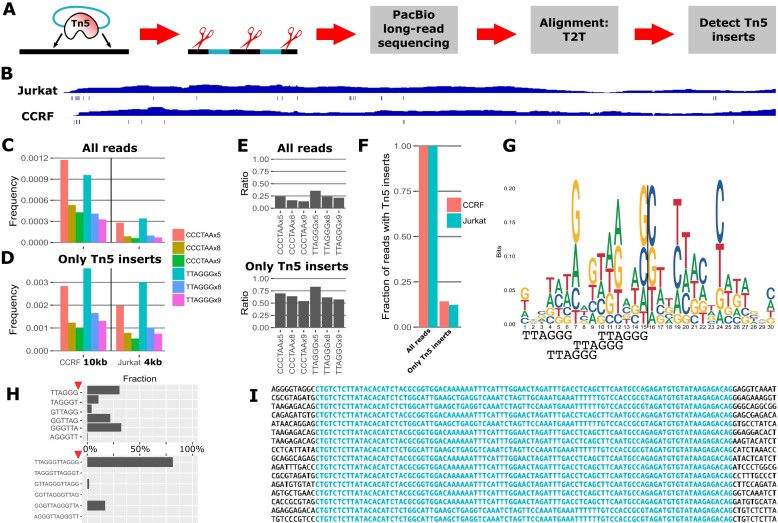
The telomere proper appears to be protected from transposition. (**A**) Experimental setup to find the precise insertion sites of Tn5 during ATAC-seq; Tn5 with nonfragmenting inserts target the genomic DNA, which is then mechanically sheared, and sequenced using PacBio. (**B**) Example coverage of the T2T genome (chromosome 1) and detected Tn5 inserts beneath. (**C**) Telomere *k*-mer-based content can estimate relative telomere lengths for cell lines of known length (i.e. the frequency is much lower in Jurkat than CCRF), (**D**) but not when Tn5-insert-containing reads alone are counted (the frequencies are similar). (**E**) The ratio of nTAs between cell lines (which should be ∼0.25) is more correct when searching for shorter telomere repeats, possibly because higher match stringency leads to loss of valid reads. (**F**) Fraction of Tn5 insertions in all fragments versus those in the telomere, indicating poor coverage transposition of the telomere proper. (**G**) Per-base motif analysis around transpositions; possible relative placements of the full TTAGGG telomeric motif, as suggested by the per-base motif, shown below. (**H**) Abundance of telomere motif near transposase inserts, showing a strong location preference that might be further reinforced by chromatin structure. (**I**) No duplication of genomic target sequence is seen, contrary to previous reports [68].

To overcome the limitations of the short-read data, we performed an experiment on two cell lines with known telomere length using long-read sequencing: CCRF (∼7.5 kb [60] and Jurkat (∼4 kb [61]). We designed the experiment (Fig. 2A) to specifically test if (i) the telomere is hypotagmented, in which case there would be too few fragments to reliably estimate the length, or (ii) hypertagmented. In either scenario, fragments would also get lost during the ATAC-seq library size selection. Hypertagmentation would further prevent precise alignment, despite access to long-read sequencing technology. To overcome these problems, we performed ATAC-seq but with a complete nonfragmenting transposon insert, requiring a separate post-transposition mechanical fragmentation. Because Nanopore library preparation itself commonly relies on a transposase, we further opted to use PacBio technology, where the input DNA is instead mechanically sheared in an unbiased manner. A large amount of cells was used to further remove the need for PCR.

The result is effectively WGS (∼12× coverage per sample; Fig. 2B), but with random Tn5 insertions. Care was taken in the analysis, as we note that Tn5 still incurred some level of fragmentation, likely from Tn5 homodimers capturing and transposing two separate, rather than one single DNA insert. Overall, a total of 1.2 M Tn5 insertion sites were detected. By computing nTA on all of the reads (i.e. as in TelSeq for WGS), Jurkat is predicted to have 14%–35% of the telomere length compared to CCRF, somewhat less than their expected telomere length ratios (Fig. 2C and E). However, if this analysis is performed only on reads having transpositions, as would be the case for standard, short-read ATAC-seq, this nTA difference disappears (Fig. 2D and E). We aligned the reads to the T2T reference genome, thus overcoming the limitations of the GRCh38 reference genome (specifically, that GRCh38 has poor coverage of repeat regions; e.g. an entire 15mb of one arm of chr13 is masked). This alignment verifies that the fraction of telomere proper reads with transpositions was lower (Fig. 2F). The source of telomere-like motifs is thus primarily from other parts of the genome when performing ATAC-seq.

To understand why the telomere appears poorly tagmented, we performed a motif analysis. It is already known that Tn5 is known to have a preference for GC-rich regions [62]. As the telomere motif TTAGGG is rich in G, it is plausible that it is tagmented. The GC bias was confirmed by our per-base motif analysis (Fig. 2G). Furthermore, when searching for inserts near or in TTAGGG motifs, we found that Tn5 prefers TTAGGG/ or TTA/GGG (Fig. 2H), in line with the motif from short-read data (Fig. 1F). This cannot be predicted solely by the per-base motif model; however, other factors affect the transposition rate as well, including the higher-order DNA structure [63], and—crucially for ATAC—any bound proteins. The telomere is also known for the presence of G-quadruplexes [64], which may further affect the transposition rate. We thus believe these factors, as part of the shelterin complex, affect the tagmentation process.

While performing the motif analysis, we also note that our per-base motif (Fig. 2G) does not match well with a previously published motif [65] beyond the GC bias. Most importantly, it has been reported that wild-type Tn5 causes a 9 bp duplication of its target [66], but we did not observe this with our hyperactive Tn5_E54K, L372P_ (Fig. 2I). It is possible that the mutations responsible for the increased activity [36] have changed this behavior. Thus, while the 9 bp duplication is widely assumed in modern ATAC-seq analysis pipelines [43,67], this may have to be revised, as ATAC-seq is always performed using a modified Tn5.

To conclude, we show that Tn5 has low affinity to the telomere proper during ATAC-seq. This partially explains why any correlation between telomere motif abundance and telomere length likely is too weak to be of practical use. For brevity, we will use the term “telomere accessibility,” or nTA, to refer to the relative number of reads in ATAC-seq having telomeric motifs, despite that the origin is primarily from the subtelomere.

### Differential accessibility of telomere-like repeat regions pinpoint broad chromatin condensation events

**Figure 3.**
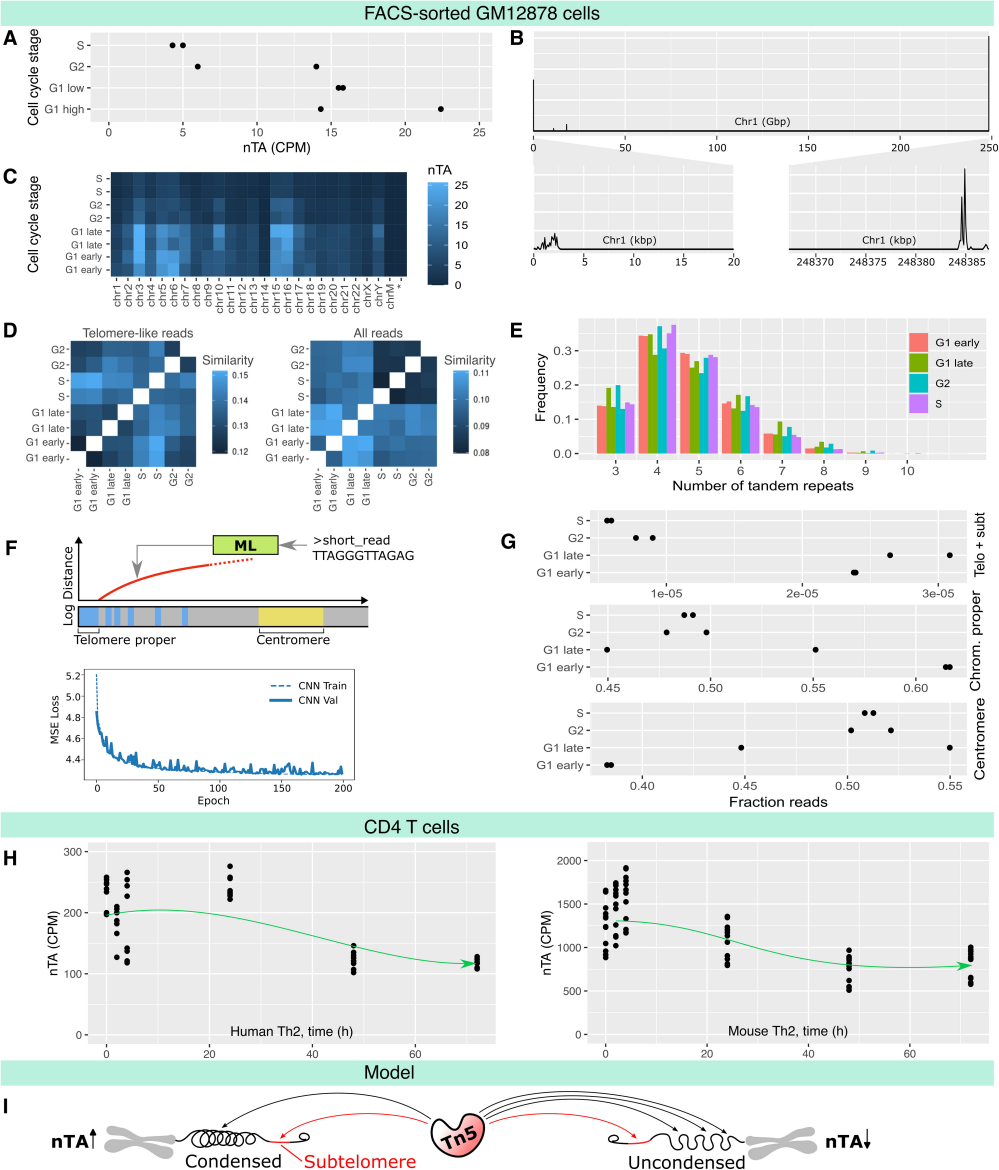
Differential accessibility of telomere-like repeat regions pinpoint broad chromatin condensation events. (**A**) Bulk ATAC-seq nTA from GM12878 cells FACS-sorted by cell cycle stage [69]. The nTA of S-phase is less than that of non-S-phase (*P*= .005). (**B**) Pileup of telomere-like reads along T2T chromosome 1, suggesting primarily a subtelomeric origin. (**C**) nTA broken down by chromosome, showing that they all co-vary similarly with cell cycle. (**D**) Similarity of bulk ATAC-seq dataset as indicated by Mash and *k*-mer similarity. (**E**) Cell cycle has little effect on the distribution of telomere motif length. (**F**) A convolutional neural network model to predict the origin of reads; MSE, mean squared error (**G**). Abundance of telomere-like reads in the different genomic regions versus cell cycle, according to the neural network model. For telomere + subtelomere, the nTA of S-phase is less than that of non-S-phase (*P*= .014). (**H**) Bulk ATAC-seq nTA in human and mouse CD4 T helper type 2 cells during the first 72 h of activation, which is a synchronized entry to S-phase. The nTA drops as expected (arrow drawn manually, conceptual only). TEM images showing the rapid chromatin decondensation have been generated previously [71]; CPM, counts per million. A linear model indicates that nTA decreases over time, for both the human (*P*= 5.1 × 10^–7^) and mouse (*P*= 1.0 × 10^–12^) time course. (**I**) The proposed model of why nTA correlates with chromatin condensation.

Telomere length inference from ATAC-seq appears infeasible given the lack of correlation with age, and lack of transpositions in the telomere proper. To further rule out the possibility of quantifying telomere length, we investigated which other factors influence the nTA measure. We reasoned that the general biological state of the chromatin may impact the tagmentation process and thus the nTA readout. The condensation during the cell cycle is an extreme case, which further allows us to compare nTA for the otherwise most homogenous cell population we can imagine. A previous study of DNA accessibility using MNase showed that the S-phase is more open than the G_1_- and G_2_-phases, while the M-phase is the most condensed [68]. We analyzed GM12878 cells, FACS-sorted for different cell cycle phases using DAPI, combined with fluorescent Tn5 inserts (“ATAC-see”), from a previous dataset [69]. It has previously been speculated that early and late G_1_-phase can be separated by the total fluorescent ATAC-see signal, which increases with higher numbers of transpositions [69]. This already shows that the cell cycle is an important driver of broad accessibility. The fluorescent signal, however, remains the same across other phases of the cell cycle. Our closer analysis shows that nTA differs little between early versus late G_1_ but is markedly low in S-phase (Fig. 3A). Aligning the telomere motif-containing reads to the T2T reference, we found that almost all of them map near the subtelomere (Fig. 3B), but with rather poor mappability. The signal appears to originate from all chromosomes similarly (Fig. 3C). This analysis, however, suffers from limitations (i.e. due to the mapping of short reads to repetitive and poorly studied regions). To circumvent this problem, we also assessed similarity between samples using the Mash metric (i.e. similarity of sequences based on overlap of min-hashed *k*-mers) [70]. Telomere-like reads have higher similarity than the genome average (Fig. 3D); but the similarity is low (<13%), suggesting a broad origin rather than a single hotspot. The S-phase telomere-like reads stand out as the most different from those of other phases according to Mash (Fig. 3D) but possibly because of their small proportion (about a quarter of the amount in other samples). The distribution of the number of telomere-like motifs in reads was not significantly different across samples (Fig. 3E).

Because the alignment of reads against short, repetitive regions is not reliable, and because motif-based analysis is rather limited, we validated the alignment and kmer-based analysis using a convolutional neural network that approximately infers the position of reads. For this purpose, we assumed the genomic model of Fig. 1B, but since the subtelomere is poorly defined, we attempted to compute the log(distance) of a read from the telomere (Fig. 3F). Furthermore, the chromatin proper and centromere were given artificially high fixed distances (see “Materials and methods”), reflecting their typical distance from the telomere. As training data, we used the T2T genome sequence for the centromere and chromatin proper. To account for the poorly studied but highly variable subtelomere and telomere regions, we obtained long-read sequencing data across 147 individuals [28]. The fitted model was then used to predict the origin of reads for the GM12878 data. As few reads were classified as telomere proper, we binned these with the subtelomeric reads. Overall, we obtained the same results and linkage to the cell cycle phase as for the *k*-mer based analysis (Fig. 3G), suggesting their equivalence and supporting the idea that the main origin of the telomere-like reads is the subtelomere. As a bonus observation, reads from the centromeric region may be an interesting biomarker for early G_1_-phase. Further study of the centromere is however saved for a future study.

To validate the dependency of nTA on the cell cycle in another cell type, we looked at the orthogonal case of human and mouse naive T cells undergoing activation. Naive T cells, until activated, remain in a condensed dormant G_1_-like state, as seen previously by the transmission electron microscopy (TEM) [71]. The activation can be seen effectively as synchronized entry into the cell cycle. In line with the GM12878 analysis, we observed that nTA drops during activation as more cells enter S-phase and proliferate (Fig. 3H). This estimate is also agreed upon using our machine learning (ML)-based estimate (Supplementary Fig. S3).

To conclude, the cell cycle has a large impact on the chromatin condensation state, and by extension, also on nTA. We note similar trends in two cell types. The origin of the telomere-like reads is broad and likely subtelomeric, as supported by alignment, and a novel long-read-informed ML model for approximate alignment. Because accessibility is a relative concept, and ATAC-see FACS showed no difference in total number of transpositions except for early G_1_ [69], we believe a low nTA score is rather due to the rest of the chromatin opening up (Fig. 3I). An alternative theory is that the telomeres are replicated at a different time point during the cycle. In yeast, the telomeres are replicated at the end of S-phase, which would be compatible; but in mammalian cells, the telomere replication is continuous [72]. In either case, it appears possible to use nTA as a biomarker for the cell cycle phase, which is the focus for the remainder of our study.

### Telomere-like read abundance can aid single-cell interpretation of chromatin condensation

**Figure 4.**
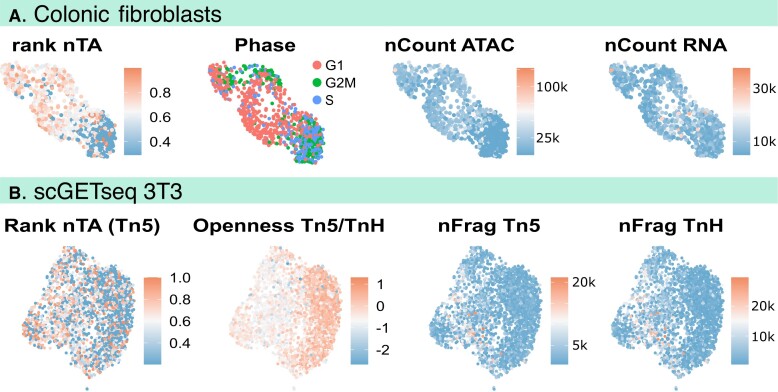
Telomere-like read abundance is sufficient for single-cell interpretation. (**A**) UMAP of a new atlas of primary colonic human fibroblasts cells (one single reaction); nTA is the lowest in S-phase, consistent with bulk measurements (Fig. 3). Quality control panels indicicate number of ATAC and RNAseq fragments. (**B**) UMAP of 3T3 cells, previously measured by scGET-seq [73]; this method measures both open and closed chromatin for direct computation of relative openness, which is negatively correlated with nTA. The panels indicate rank nTA, the openness defined as the ratio of Tn5 to tnH fragments, number of Tn5 and TnH fragments.

To see if nTA can aid in single-cell data interpretation of chromatin state, we focused on two datasets in which the cell cycle is an expected major driver for the clustering. First, we generated a new single-cell multiome dataset of primary colonic fibroblasts, as previous RNA-only fibroblast atlases have determined that cell cycle is the major driving component [16]. We note that the S phase cells are clearly separated, and that nTA is lowest among these cells, as expected from our postulated model (Fig. 4A).

An alternative measure of openness is provided by scGET-seq, a single-cell technology in which normal Tn5 is combined with a modified Tn5 that targets H3K9me3, which decorates the heterochromatin. Thus for each cell, the ratio of open and closed chromatin can be computed, and the ratio is lower in mitotic cells [73]. In 3T3 cells, we found a negative correlation between openness (tn5/tnH) and nTA (ρ = –0.12, *P*< 3 *×*10^–10^, Fig. 4B), i.e. high openness means low nTA. The previous analysis of this dataset also suggested that the low-openness cells are mitotic [73], in line with nTA being linked to the cell cycle.

To conclude, the cell cycle phase is a strong driver of nTA, and this is especially clear in simple cell types (i.e. where other types of heterogeneity is low). We have, however, assayed most public multiome single-cell data to date (the nTA for all datasets analyzed is available in Supplementary Data), and we have observed other correlations that are harder to untangle. An example is mouse embryogenesis (Supplementary Fig. S5), where nTA still correlates with cell cycle, but likely also cell type. nTA must thus be interpreted within the context of other markers to be truly informative.

### nTA complements RNA-based cell cycle analysis

**Figure 5.**
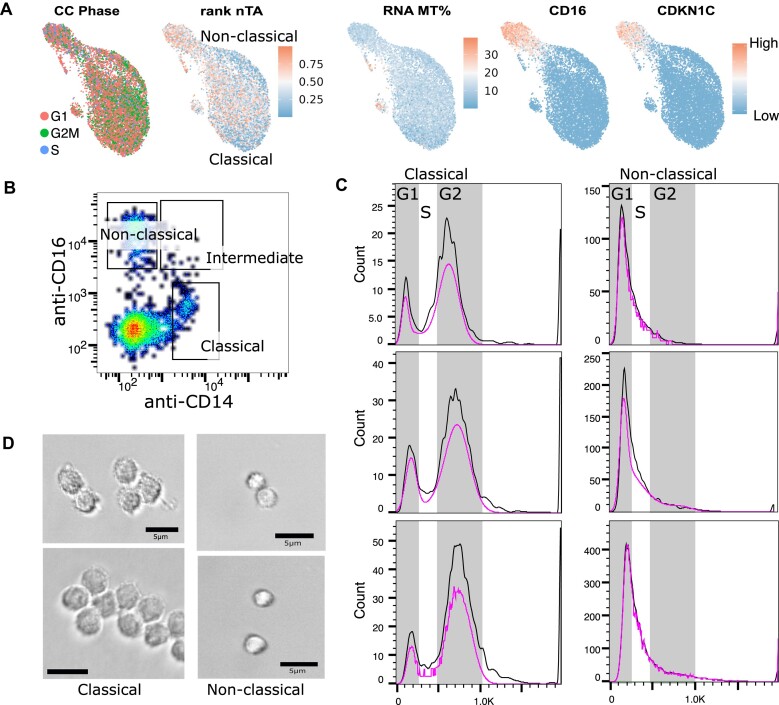
Comparison of monocyte telomere accessibility versus cell cycle. (**A**) UMAP of monocyte cells in the 10X Genomics PBMC multiome dataset, showing the nTA, markers for subsets, and RNA-based prediction of cell cycle phase. Based on the possible elevation of nTA in the nonclassical subset, we performed further analysis of condensation. CDKN1C is one of the most correlating genes with nTA that is likely to explain condensation in terms of cell cycle. (**B**) FACS gating for the sorting of different monocyte populations. (**C**) DAPI staining of monocyte populations to determine cell cycle phase distribution. (**D**) Images of monocyte populations, where classical monocytes appear bigger and more grainy than the nonclassical subset.

To see if nTA is useful for making predictions, we reanalyzed existing public single-cell multiome data. We found a human PBMC dataset from 10× Genomics (25 000 cells), which contains monocytes. These are especially interesting from a chromatin condensation perspective, as they are generally characterized by bilobed, horseshoe-shaped nuclei, which we figured might have a strong impact on chromatin accessibility patterns. However, there are multiple monocyte subsets, with different functions [74]. In this single-cell dataset, nonclassical CD14^lo^CD16 + monocytes have a higher nTA (Fig. 5A, quality metrics in Supplementary Fig. S4B and C). A potential explanation is that the second-most positively nTA-correlated gene is CDKN1C (24%), which inhibits proliferation during the G_1_-phase [75]. We verified using FACS that this subset indeed is stalled in G_1_ (Fig. 5B and C), and also has different morphology, with nonclassical monocytes having less granules (Fig. 5D). Interestingly, typical RNA-seq-based cell cycle annotation thus generates the wrong prediction (Fig. 5A).

We also investigated if the cell cycle can be studied via the TERRA transcript [76]. TERRA is transcribed from the telomere and subtelomere, and is used as a template during telomere elongation. In several cell lines, TERRA peaks in G_1_-phase, dropping over S-phase [77]. We, however, find the abundance of TERRA reads to be extremely low in 10× genomics RNA-seq data (Fig. 1E), and we have generally not seen appreciable TERRA fractions in any public 10× Genomics RNA-seq dataset. This is likely because only 7% of human TERRA transcripts are polyadenylated [78]; this poses a problem for most single-cell RNA-seq protocols as they use oligo-dT for reverse transcription. A rare exception is that of Parse Biosciences WT v3, which also includes random hexamers. We analyzed a public Parse biosciences PBMC dataset and found sufficient abundance of TERRA for analysis (Supplementary Fig. S6). Interestingly, we find TERRA to be down in the CDKN1C nonclassical monocytes (Supplementary Fig. S6D and E), contrary to previous studies in cell lines [77]. It thus does not seem that a naive analysis of TERRA can be used to infer cell cycle phase. There are several layers of regulation that current scRNA-seq struggle to account for; e.g. polyA- and polyA+ TERRA have different function [77] (only polyA- is associated with chromatin), and these cannot easily be separated in the RNA-seq data. Another oddity of the Parse Biosciences protocol is that it does not suffer from major rRNA inclusion, despite the use of random hexamers and having no rRNA (ribosomal RNA) depletion; we know no explanation for this except that it might be due to secondary structures selectively preventing RT. Also, TERRA can form G-quadruplexes under certain conditions [79]—this not only prevents enzymatic degradation but also prevents RT [80]. Thus, major further work is required to assign an interpretation to TERRA-like reads in scRNA-seq data. Those interested may however wish to investigate the Parse Biosciences protocol combined with long-read sequencing to obtain more conclusive data.

To summarize, the monocyte example highlights the difficulty in precisely annotating the cell cycle phase from RNA-seq data. Notably, at least 16 different bioinformatics tools/methods exist for this purpose to date [81]. While we do not claim general better performance over RNA-seq-based cell cycle condensation, we do however postulate that ATAC-seq provides orthogonal data, and that chromatin condensation/decondensation needs to happen regardless of which (potentially cell type specific) genes are regulating the process. Thus, multiome analysis might be a better choice than RNA-seq analysis on its own, for applications when the cell cycle state is of particular interest.

### ATAC-seq across tissues and cell types pinpoints transcription factors linked to chromatin condensation

**Figure 6.**
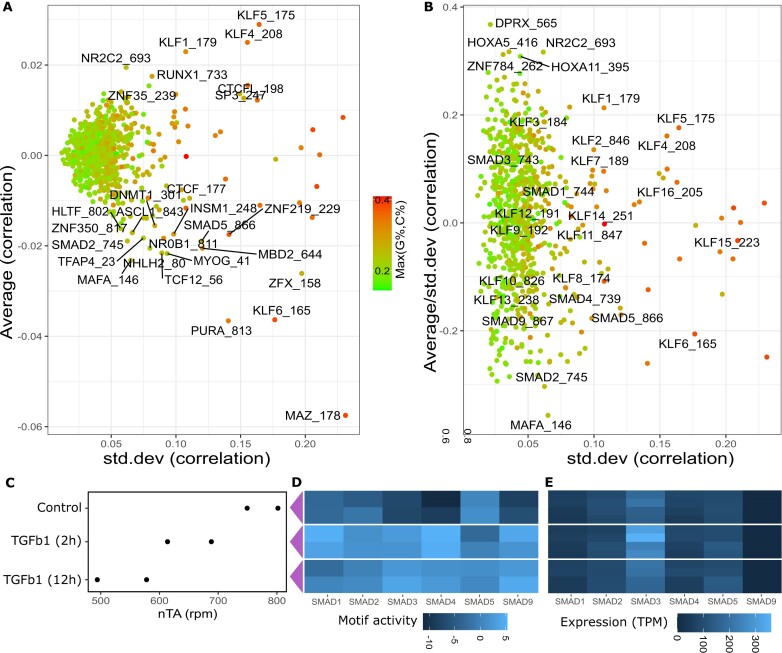
ATAC-seq across tissues and cell types pinpoints TFs linked to chromatin condensation. (**A**) Mean and variance of correlations of motifs versus nTA, across cells from a previous whole-body atlas [21]. (**B**) Normalized mean correlation removes GC effect on variance. (**C**) nTA across epithelial cells treated with TGFb fits the microscopy-validated chromatin opening [84]. (**D**) Analysis of SMAD motif activities during treatment. (**E**) Expression of corresponding genes Smad1-9 from RNA-seq. Smad9 is on average < 1 TPM (transcripts per million), all other at least 50 TPM.

As we expected nTA to also be driven by cell type-specific factors, and not just the cell cycle, we reanalyzed a recent sci-ATAC-seq atlas of chromatin accessibility in the human genome [21]. The nTA of the publicly available subset (500k cells) was computed for different cells across tissues in (Supplementary Fig. S7, nTA values in Supplementary File S1). Naive T cells have a high nTA, reflecting previous observations of their condensed state. To find the most common drivers of nTA, we computed correlations for each cell type and batch (only keeping subsets with >1000 cells to reduce noise). The average correlation of each motif is shown in (Fig. 6A). Some of the most highly correlating motifs (Supplementary Fig. S8C) include the KLF (Krüppel-like factor) family. KLF4 has been linked to the G_1_–S transition [82] and TERT expression [83]. However, on a closer inspection, there is also a higher variance (Fig. 6A) for motifs with high G% or C%. As the telomeric repeat has a high G content (TTAGGG, 50%), these correlations might be artificially inflated, though there is no correlation *per se* between GC content and correlation (Supplementary Fig. S8A and B).

To account for the potentially confounding effect of GC%, we instead ranked motifs by the mean/variance ratio (Fig. 6B), which largely removes the GC bias and de-emphasizes the KLF family. Instead, among the most correlating TFs which we could validate with public data, we found the SMAD family (SMAD2 being one of the most correlated motifs). As SMAD is downstream of TGFb, we reanalyzed a previous study of TGFb (transforming growth factor beta) in NMuMG epithelial cells [84]. This is also one of few studies including ATAC-see, and it shows that TGFb leads to global chromatin opening, abbrogated by siRNA (small interfering RNA) against Smad4. The chromatin opening is consistent with the nTA (Fig. 6C). As a further validation, we investigated the SMAD motif activity. A complication is that Smad4 is a cofactor, typically forming a heterodimer with Smad2 or Smad3 [85]. While chromVAR is designed for single-cell analysis, we deviced a package to aggregate bulk samples into a format compatible with Signac, and thus also chromVAR (see “Materials and methods” section). The chromVAR suggests (Fig. 6D) that all SMAD motifs increase in activity except for SMAD5; this is however likely an artifact, as chromVARs struggle to fit motifs that are similar (Supplemental Fig. S8D), especially with limited number of datapoints. This is especially clear from Smad9, which has differential motif activity, despite not being expressed (Fig. 6E). While the previous study proves that SMAD regulates chromatin opening, it also shows that the motif correlations must be interpreted qualitatively given the limitations in chromVAR.

To summarize, correlation with motif activities, as computed using standard methods, suggests some TFs that might be regulators of chromatin condendations. The SMAD family has been validated to regulate condensation [84] and this is implicated in our analysis. The KLF family might also be involved, but it is at this point unclear if the influence of motif GC richness is biologically linked or is a technical artifact. For those interested in further investigation of different cell types, we provide precomputed nTAs for cells across the entire body as a resource for further exploration (Supplementary Data).

### ATAC-seq implicates putative drivers of chromatin condensation during B-cell somatic hypermutation

**Figure 7.**
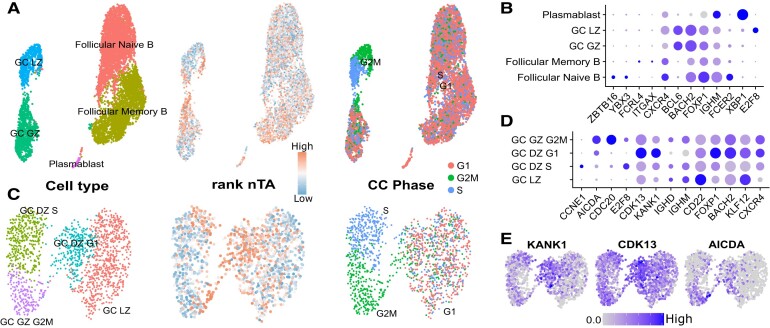
A multiome atlas of tonsillar human B cells. (**A**) UMAP of all B cells. (**B**) Expression levels of representative marker genes for all B cells. (**C**) UMAP of germinal center B cells only. (**D**) Expression level of representative marker genes for the germinal center B cells. (**E**) Expression of AICDA and some of the genes most correlating with nTA.

As an example application of how the nTA measure can be used for hypothesis generation in a typical single-cell setting, we generated and applied our measure to an atlas of human tonsillar B cells across 5 individuals (Fig. 7A and B and Supplementary Fig. S8A and B). B cells are the source of antibodies, which evolve in the lymph node germinal center through two modes of genome self-editing: Somatic hypermutation (SHM) and class switch recombination (CSR). As off-target mutations can lead to cancer, this process is tightly controlled [86]. It has been shown through microscopy that the mutagenic enzyme AICDA (activation-induced cytidine deaminase) is present in cytoplasm and only enters the nucleus during cell division, likely as a titration mechanism. Thus, mutagenesis happens during early G_1_-phase [87]. We speculate that the simultaneous chromatin condensation also promotes selective mutagenesis of the B-cell receptor, and that single-cell analysis of the condensation process may aid in finding regulators of this process.

Our atlas covers both follicular and germinal center (GC) B cells (Fig. 7A). For the purpose of this analysis, we however focused on the GC B cells only, where SHM/CSR occurs (Fig. 7C and D and Supplementary Fig. S8C and D). These cells are primarily separated not only along the light/dark zone gradient (CD83-CXCR4) but also by cell cycle. This is in line with classical models of SHM/CSR and expansion happening in the dark zone [88]. The gene AICDA, responsible for SHM/CSR, is especially expressed among cells in the G_2_/M-phase, extending into G_1_-phase. Similarly, nTA does not align with the RNA-based cell cycle annotation but has a similar pattern (Fig. 7A, C, and E). Overall, while we cannot exclude other drivers of nTA than condensation, these observations are all consistent with the observation that AICDA acts in early G_1_, when the chromatin is condensed [87], and thus must be expressed at some point before G_1_ (i.e. G_2_/M). The level of nTA is also consistent with this idea and may thus plausibly reflect the level of condensation.

Based on this assumption, we used correlation to find putative drivers of condensation (Supplementary File S3). The top-most correlating genes are the TF FOXP1 (Forkhead box protein, 25%, *P*< 2.5e-24), followed by the TF ZNF831 (25%, *P*< 1.9e-23) (Supplementary Fig. S9). FOXP1 has previously been linked to B-cell lymphoma [89] while ZNF831 is overall poorly studied. Other correlating genes include the actin polymerization regulator KANK1 (KN Motif And Ankyrin Repeat Domains 1, 22%, *P*< 1.1e-20, Fig. 7E), for which KO can lead to cell proliferation [90]. nTA also correlates with CDK13 (Cyclin dependent kinase, 23%, *P*< 8.6e-20, Fig. 7E), which has been shown to increase Pol II processivity [91]. GWAS (Genome-wide association studies) have associated CDK13 with the amount of IgD + CD38^dim^ and IgD + CD24- B cells [92], or lymphocyte count in general [93, 94]. It is therefore possible that CDK13 upregulates DNA damage response genes, as previously shown for CDK12 [95], and thereby helps control the dose of SHM. Further validation of these genes is however outside the scope of this manuscript.

Overall, our analysis shows that nTA, for a specific cell type, might reflect known chromatin dynamics. As in the monocyte example, the RNA-based cell cycle annotation might not be the optimal, especially if the chromatin state is of primary interest. nTA can then instead be used to infer putative regulators, such as CDK13, which can aid in understanding how lymphoma may form due to AICDA off-target mutations.

## Discussion

In this study, we initially aimed to develop a way to infer telomere length by quantifying the presence of telomere-like reads in ATAC-seq data, similar to previous WGS-based approaches. By generating a long-read atlas of transpositions, we conclude that the telomere appears largely protected from transposition, resulting in a signal too weak for detection. As we thought it might still be possible to improve the signal-to-noise by accounting for technical confounding factors, we set out to find such confounders and establish an interpretation of the telomere-like reads. We find the cell cycle to be especially relevant, as differential accessibility to the subtelomere versus the remainder of the genome affects the normalized telomere abundance score (nTA). Other quality metrics for ATAC-seq are also affected, such as reads/cell, or nucleosome signal, but we do not find consistent correlations that can be used for compensation in, e.g. a linear model for telomere length. This is still the case when accounting for batch differences, which may be caused by for example different tagmentation times and SPRI (solid-phase reversible immobilization) bead cleaning ratios (size section). Based on our exhaustive analysis, telomere length prediction at the single-cell or bulk level is not viable from ATAC-seq.

Our long-read sequencing of Tn5 inserts suggests that the telomere-like reads are of subtelomeric origin. However, this interpretation of nTA is based on the classical model in which the telomere is a rather pure TTAGGG_n_ repeat (“telomere proper,” Fig. 1B). We chose this definition to simplify the narrative, but a more modern view of the telomere may include, e.g. TVRs. Similarly, the subtelomere is poorly defined; it is rather a continuum that comprises ∼25% of the most distal 500 kb and 80% of the most distal 100 kb in human DNA [96]. Telomeres are also of different conserved lengths for each chromosome [28], suggesting that each arm is regulated differently. Here, we only consider the average subtelomeric read, being limited by short-read sequencing technology. Thus, nTA is a crude biomarker, which likely can be refined in future work. Our alternative approach of predicting the origin of reads using convolutional neural networks is promising but requires higher quality training data to reach its full potential.

One further possible driver of nTA, that we have not analyzed in depth, is the telomere position effect. Genes close to the telomere arms are selectively silenced through interaction with the telomere, which can interact with gDNA up to 10 mb from the ends [97]. The silencing reduces with age as the telomere is shortened, with ∼200 to 1600 genes significantly affected in a tissue-dependent manner [98], and this may subsequently affect subtelomere accessibility. We do not find significant correlations in our models (Fig. 1C) in support of this effect. This may be because only 1–8% of the chromatin is affected by the telomere position effect, compared to all of the chromatin for the cell cycle [99]; but also because of low sample size and high batch variation [25].

Accepting that the nTA score appears primarily linked to chromatin condensation, in a cell type-specific manner, we however show that it can still be used to help interpret single-cell data. We provide a tool, Telomemore, for the single-cell quantification of mainly-subtelomeric TTAGGG-containing reads. Using monocytes as an example, we find that RNA-seq-based cell cycle inference is not always reliable. However, it appears that nTA can be used as a complement for cell cycle interpretation. Using a large number of different cell types, we further find TFs that appear relevant for chromatin condensation, including the SMAD family that has previously been validated using microscopy (ATAC-see). Finally, we use the nTA measure to study the closing down of the chromatin during B-cell SHM, which is crucial to avoid off-target mutations and B-cell lymphoma. Correlation with nTA suggests that the cell cycle regulator CDK13 may be an important driver for this process.

Further interesting observations were made during this study; notably, the wide assumption that Tn5 duplicates 9 bp of the target region appears wrong, at least for the mutated hyperactive Tn5 used for ATAC-seq. ATAC-seq reads of the centromere may also be informative to pinpoint the early G_1_-phase, but more work is needed to validate this correlation. Telomemore can possibly also be used to study the TERRA transcript levels in Parse Biosciences scRNAseq data, as it includes random hexamers in the reverse transcription. The read abundance is however still low, and there is as-of-writing insufficient public data for a deep analysis of TERRA.

The production of this manuscript highlighted the need for the FAIR principles (Findability, Accessibility, Interoperability, and Reuse). Much single-cell data have only been made available as processed count tables, with the implicit assumption that it contains all interesting information. However, others have made use of mtDNA to perform lineage tracing [100], and we provide further uses of the raw data. We thus urge others to always release their sequencing data as raw FASTQ files, and ideally also deposit it in the Human Cell Atlas to aid reprocessing. Finally, this study would not have been possible to conduct without easy access to primary data, and a significant amount of effort went into obtaining data access agreements. Thus, we call for a discussion of which data should be considered “sensitive,” as needlessly hiding raw data slows down research and is against the interest of those benefiting from drugs derived from the analysis of human single-cell data.

## Supplementary Material

gkaf031_Supplemental_Files

## Data Availability

All sequencing data generated in this study have been uploaded to ArrayExpress (human tonsillar multiome B-cell atlas at E-MTAB-12632, multiome fibroblast atlas at E-MTAB-14220, and long-read transposed DNA at E-MTAB-14238). Telomemore is an open source and the latest version is freely available at Github, https://github.com/henriksson-lab/telomemore.java. A frozen version of this code, along with scripts to reanalyze the various datasets and precomputed nTA abundances, is deposited at Zenodo: https://doi.org/10.5281/zenodo.14278902. The single cell data can be viewed interactively at http://data.henlab.org/.
