# Supplementary material for: Telomemore enables single-cell analysis of cell cycle and chromatin condensation
Source: Nucleic Acids Res. 2025 Jan 29;53(3):gkaf031. doi: 10.1093/nar/gkaf031 (PMC11775621; doi:10.1093/nar/gkaf031)
Supplement: gkaf031_Supplemental_Files [file gkaf031_supplemental_files.zip › all sup figures.pdf]

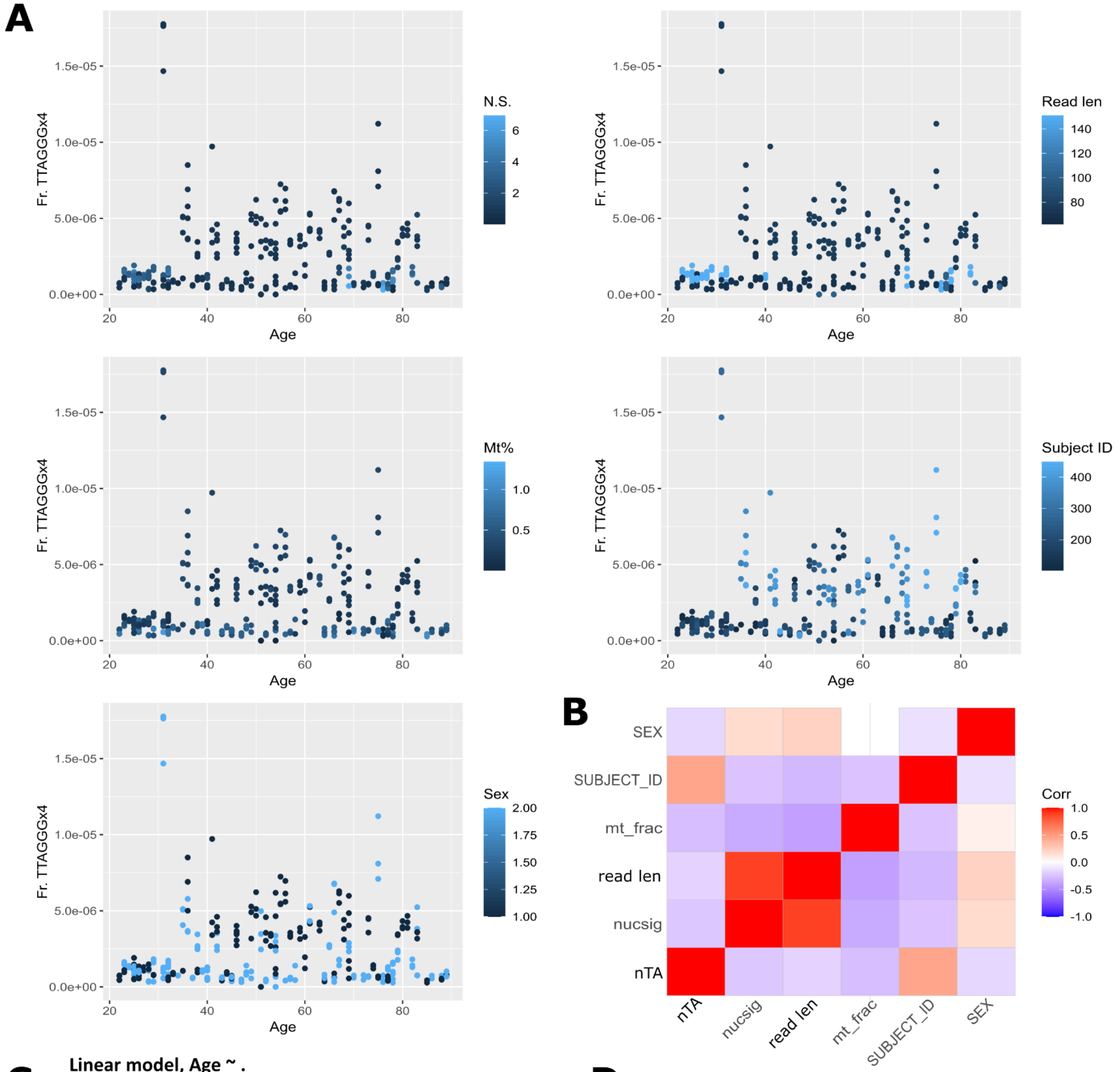

C

Linear model, Age ~ .

	Coef	DF	P(>F)
**kmer telo fraction**	-4.584e+05	1	0.3826
**Mt%**	-8.179e+02	1	0.1217
**BatchB**	7.289e+00	3	4.656e-13
**BatchC**	-4.337e+01		
**BatchD**	5.640e+00		
**N.S.**	1.029e+01	1	1.306e-9
**Sex**	1.283e+00	1	0.5374

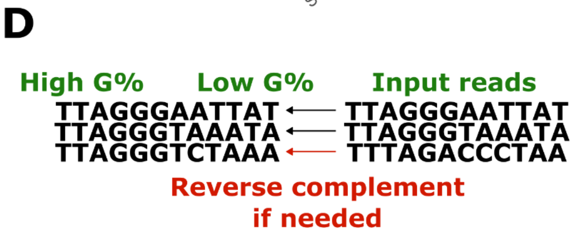

Supplemental Figure 1: Analysis of confounders in PBMC age prediction. (a) *Data points colored by possible confounding variables (Nucleosome signal, Mitochondrial % of reads, Sex, Average read length, Subject ID).* ***(b)*** *Correlation of QC parameters to telomere-like read counts.* ***(c)*** *Example linear model, trying to regress out confounders.* ***(d)*** *Reference-free alignment of reads for the purpose of motif construction.*

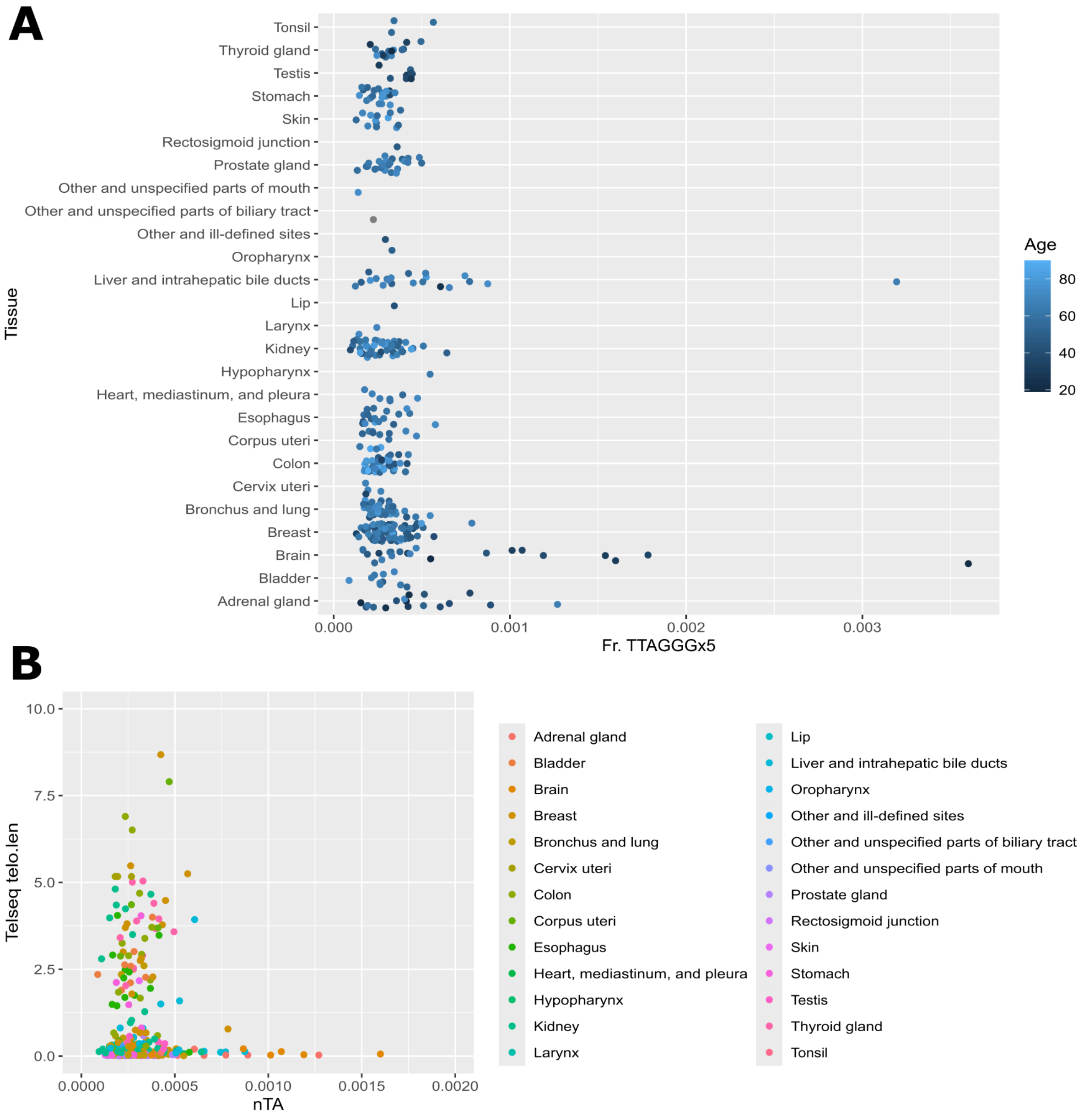

Supplemental Figure 2: Analysis of confounders in TCGA telomere length prediction. (a) *Data points separated by tissue origin.* ***(b)*** *Scatter plot of nTA vs estimated telomere length, colored by cancer type.*

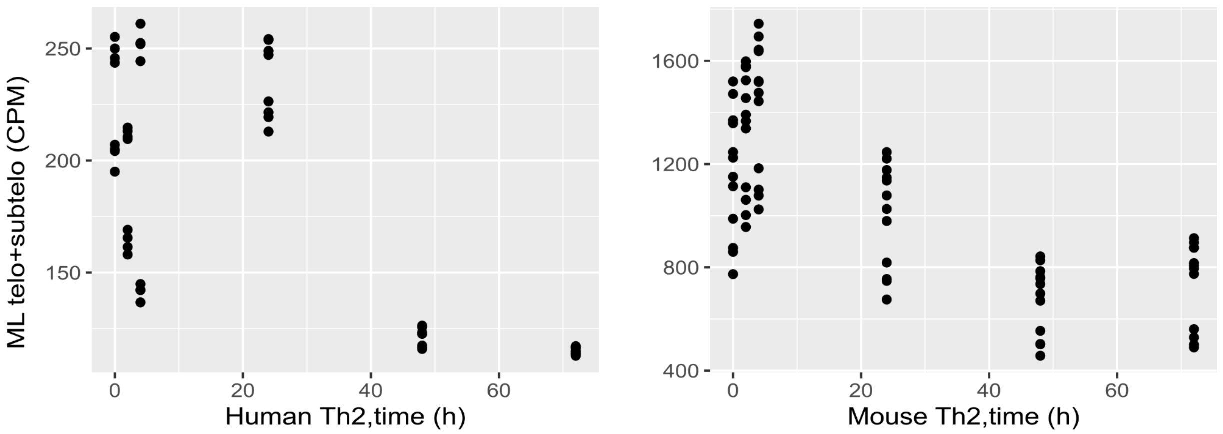

Supplemental Figure 3. Machine learning (ML)-based analysis of subtelomere abundance in mouse Th2. *Bulk ATAC-seq nTA in human and mouse CD4 T helper type 2 cells during the first 72h of activation. The predictions of subtelomere abundance, using the convolutional neural network (Fig 3f), are similar to k-mer based estimates. A linear model indicates that nTA decreases over time for both the human ($p=2.9\times10^{-8}$) and mouse ($p=7.0\times10^{-13}$) time course.*

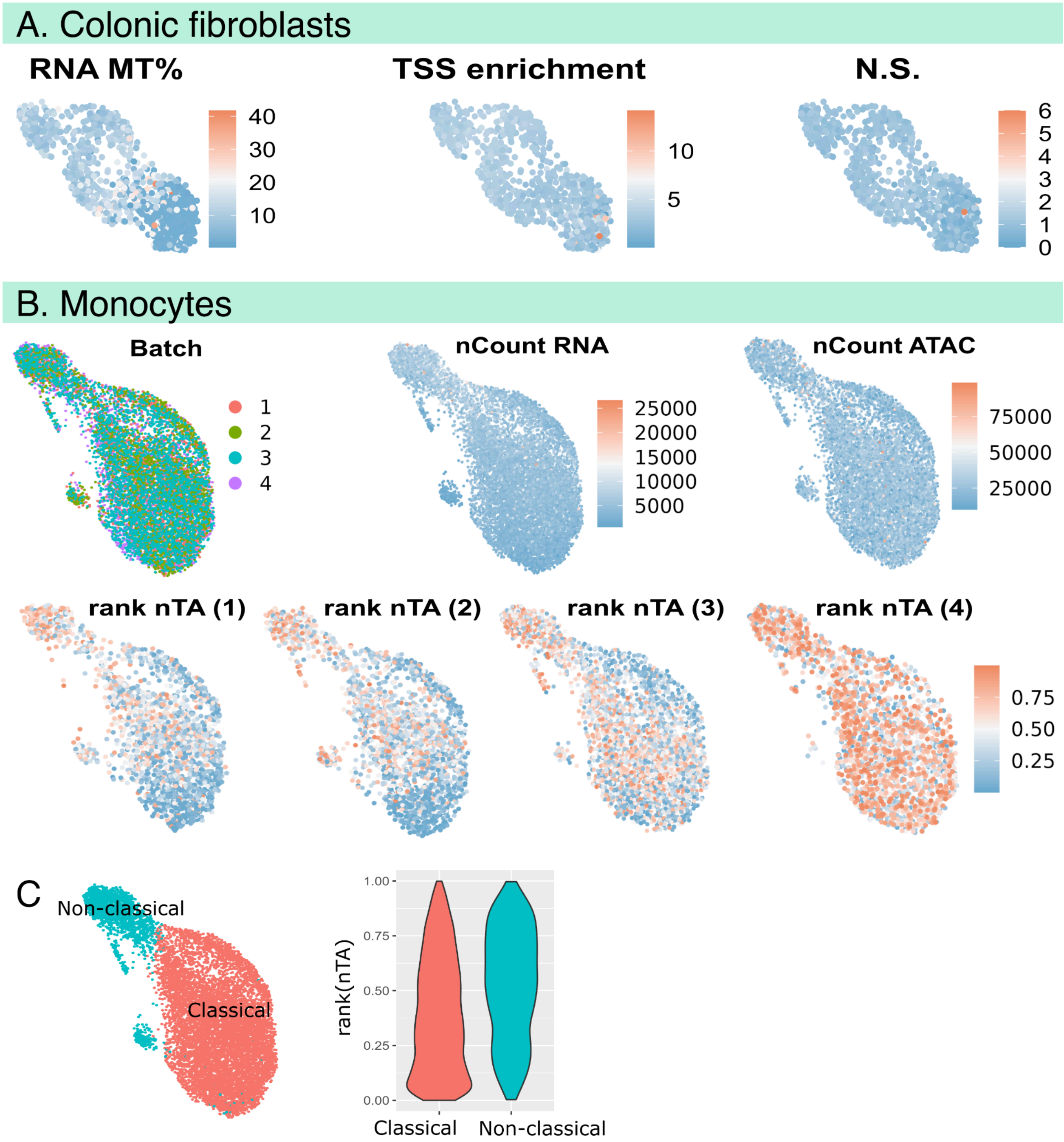

Supplemental Figure 4. Further quality control measures of multiome datasets (a) *Quality metrics for the multiome atlas of primary colonic human fibroblasts. The panels indicate RNA mitochondrial % of reads, TSS enrichment and nucleosome signal.* ***(b)*** *Quality metrics for the monocytes in the PBMC multiome atlas, including the mixing of cells across batches, the number of RNAseq and ATACseq reads. Rank nTA is shown for each of the four batches.* ***(c)*** *Average nTA for each subtype, with subtypes defined by clustering.*

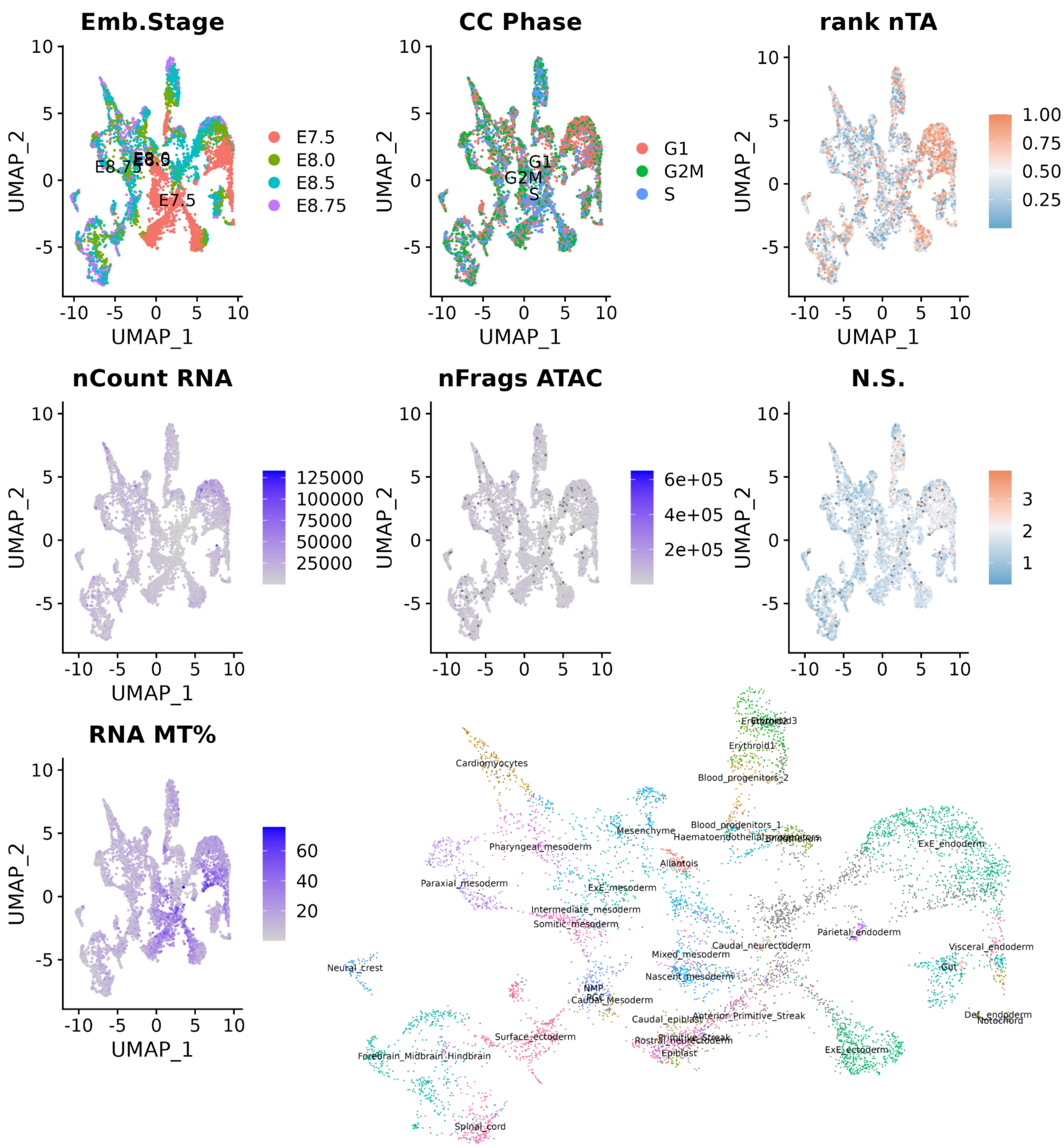

Supplemental Figure 5. A complex example of nTA in mouse embryogenesis. *Multiome data from a previous study* ***(1).*** *There is a strong overlap of G1 phase cells with a region of high nTA, as expected from other results in this study. However, nTA is also high in other locations, suggesting the existence of cell type-specific and other contributions to nTA. Nucleosome signal does not correlate well with nTA and is thus not an equivalent measure. The cells are separated across multiome runs in a non-random manner, making unbiased comparison difficult. Rank nTA is computed per-batch, which will overcorrect differences between the 8 batches, organized by stage.*

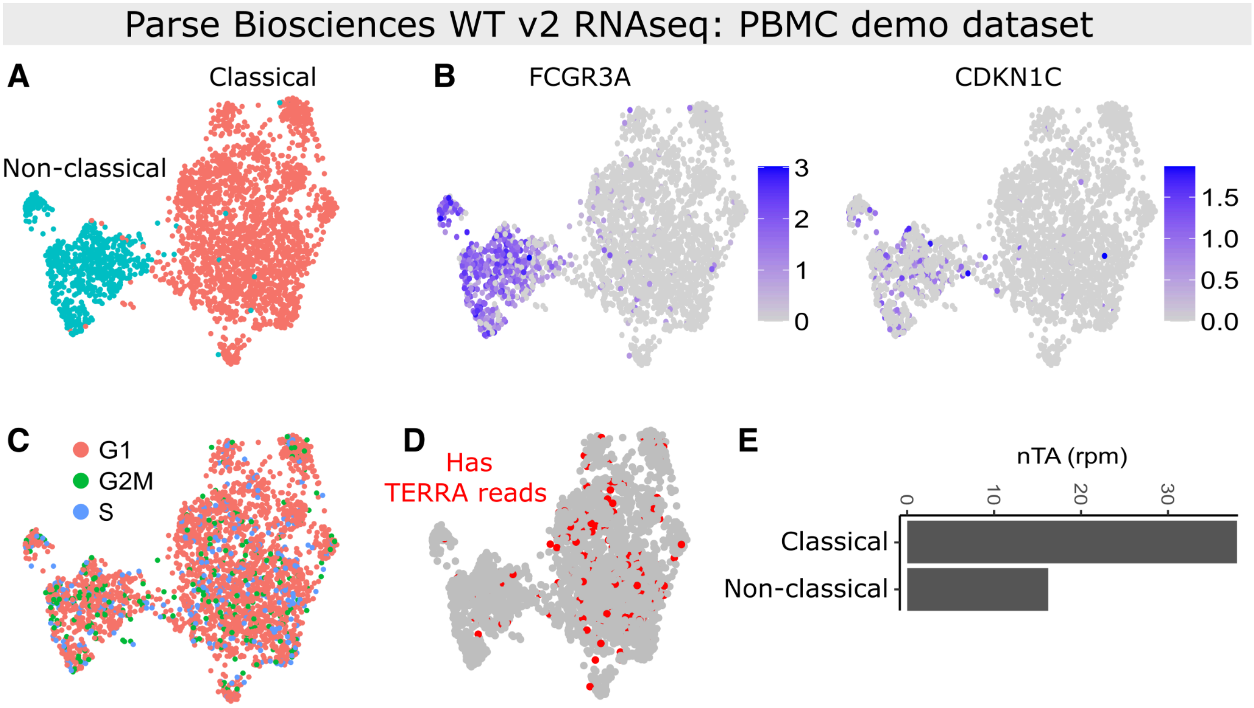

Supplemental Figure 6. Analysis of TERRA in monocytes. ***(a)*** *Corresponding monocyte clusters for monocytes in Parse biosciences PBMC RNA-seq dataset.* ***(b)*** *Marker genes.* ***(c)*** *Cell cycle inference fails also for this dataset.* ***(d)*** *Cells having TERRA reads.* ***(e)*** *Comparison of TERRA abundance between the two clusters.*

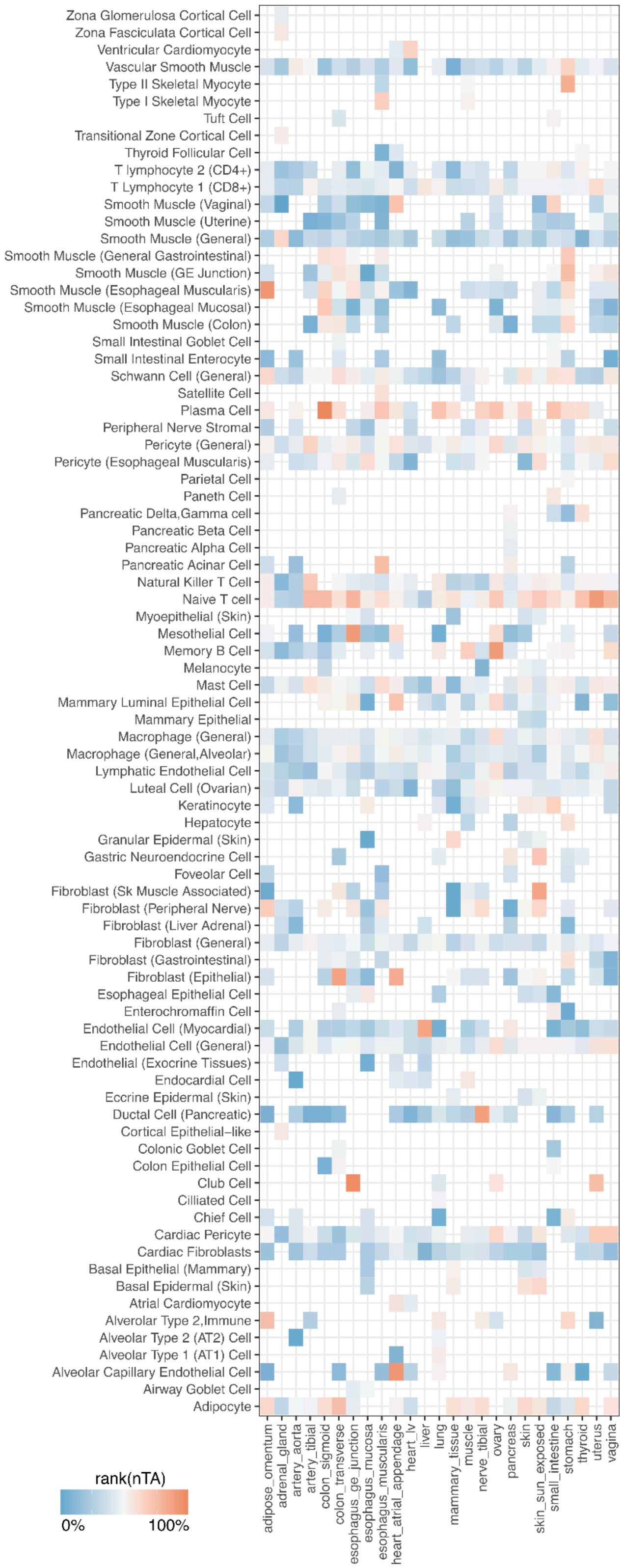

Supplemental Figure 7. A single-cell atlas of telomere accessibility in the human genome. *Normalized telomere accessibility across human cells and tissues (2).*

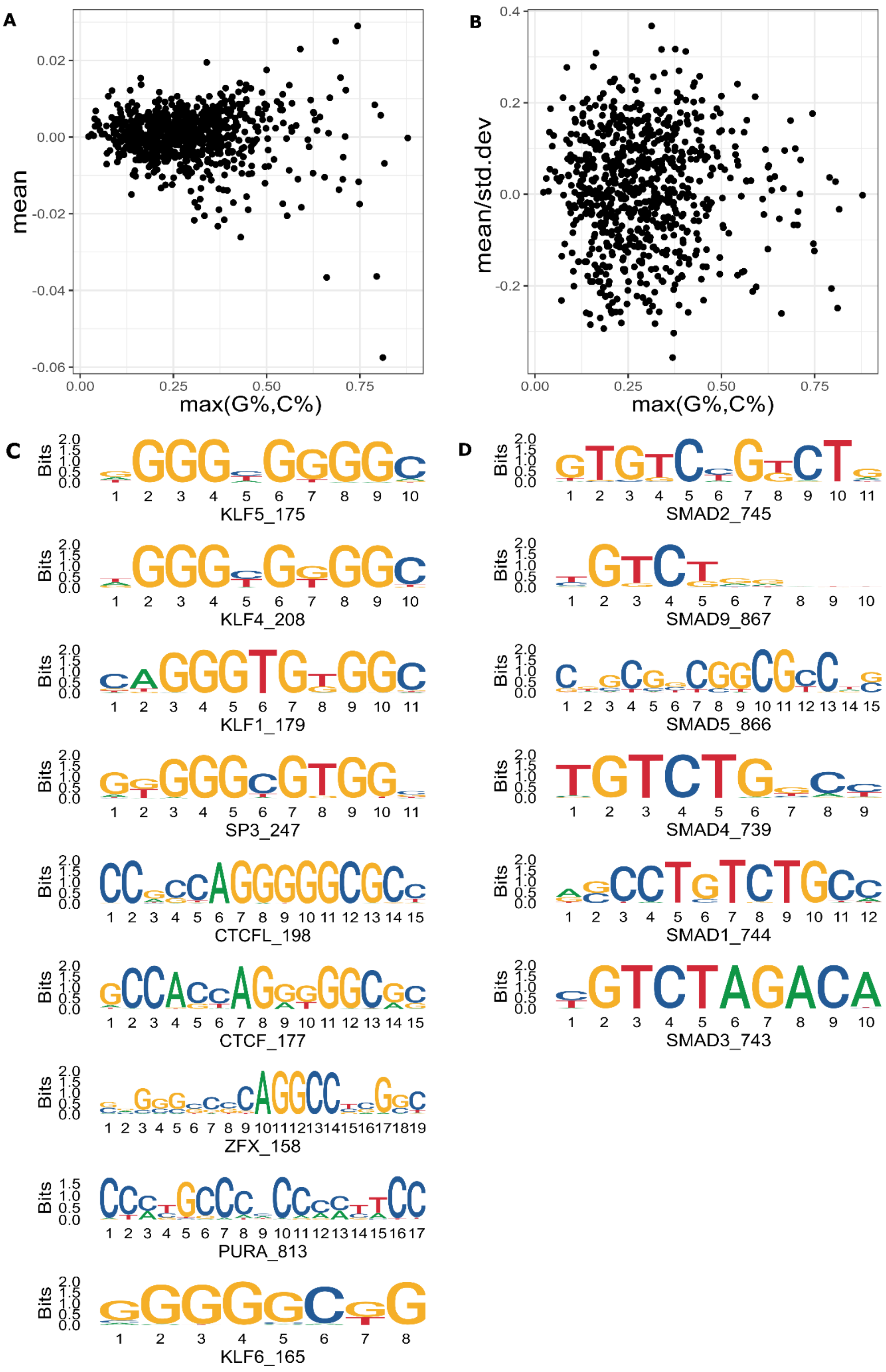

Supplemental Figure 8. Detailed analysis of nTA-correlated motifs (a-b) *Unlike the variance, The mean correlation, and normalized mean correlation, is not affected by GC content of the motif.* ***(c)*** *Some of the top correlating motifs based on mean correlation.* ***(d)*** *Similarity between the SMAD family motifs.*

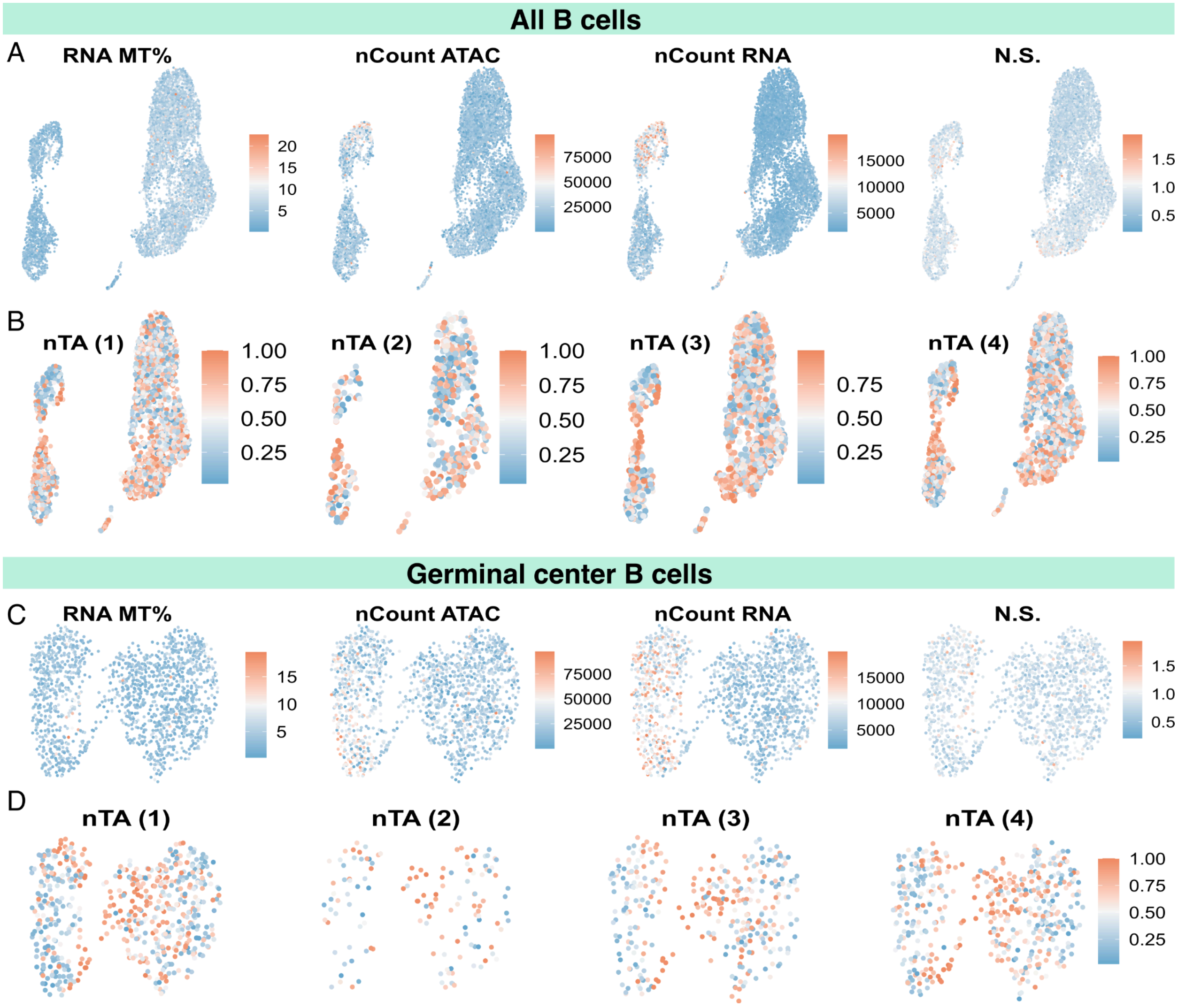

Supplemental Figure 8: Quality control of the B cell atlas. (a) *General QC measures for all B cells; N.S - nucleosome signal.* ***(b)*** *nTA per donor* ***(c)*** *General QC measures for the germinal center cells in particular.* ***(d)*** *nTA per donor for the germinal center B cells.*

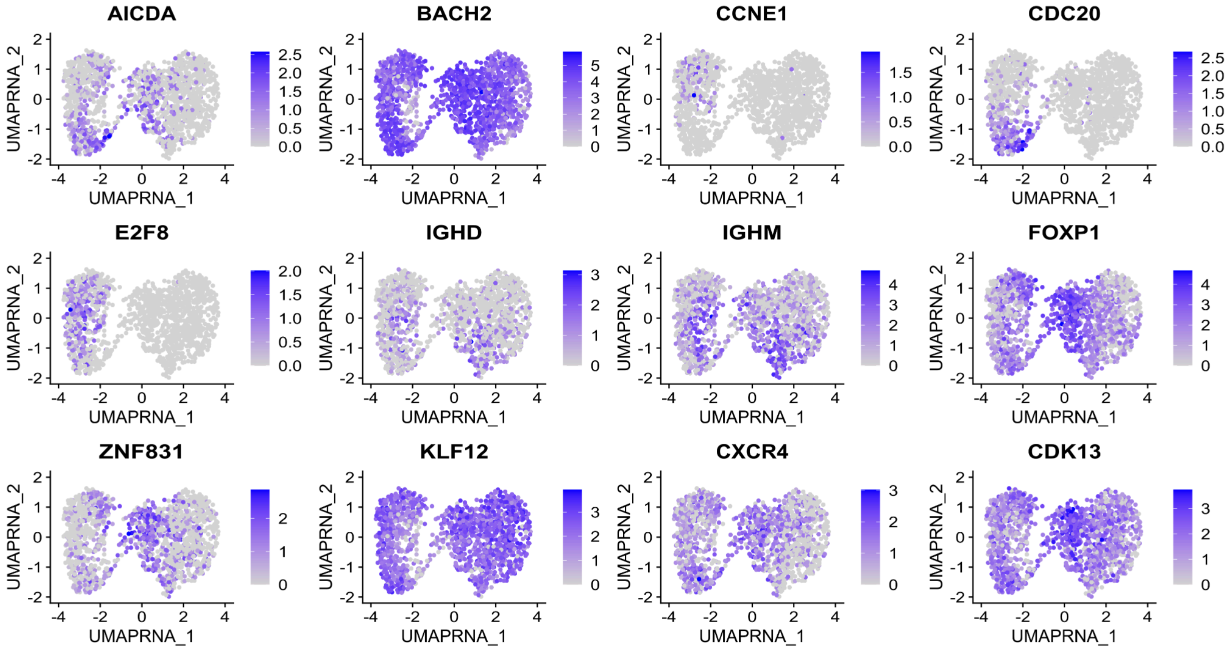

Supplemental Figure 9. Detailed expression patterns of some germinal center B cell marker genes.

SUPPLEMENTAL REFERENCES

1. Argelaguet,R., Lohoff,T., Li,J.G., Nakhuda,A., Drage,D., Krueger,F., Velten,L., Clark,S.J. and Reik,W. (2022) Decoding gene regulation in the mouse embryo using single-cell multi-omics. *bioRxiv*, 10.1101/2022.06.15.496239.

2. Zhang,K., Hocker,J.D., Miller,M., Hou,X., Chiou,J., Poirion,O.B., Qiu,Y., Li,Y.E., Gaulton,K.J., Wang,A., *et al.* (2021) A single-cell atlas of chromatin accessibility in the human genome. *Cell*, **184**, 5985–6001.e19.
